# *In silico *identification of the sea squirt selenoproteome

**DOI:** 10.1186/1471-2164-11-289

**Published:** 2010-05-10

**Authors:** Liang Jiang, Qiong Liu, Jiazuan Ni

**Affiliations:** 1 Changchun Institute of Applied Chemistry, Chinese Academy of Sciences, Changchun 130022, PR China; 2 College of Life Sciences, Shenzhen University, Shenzhen, 518060, PR China; 3 Graduate University of the Chinese Academy of Sciences, Chinese Academy of Sciences, Changchun 130022, PR China

## Abstract

**Background:**

Computational methods for identifying selenoproteins have been developed rapidly in recent years. However, it is still difficult to identify the open reading frame (ORF) of eukaryotic selenoprotein gene, because the TGA codon for a selenocysteine (Sec) residue in the active centre of selenoprotein is traditionally a terminal signal of protein translation. Although the identification of selenoproteins from genomes through bioinformatics methods has been conducted in bacteria, unicellular eukaryotes, insects and several vertebrates, only a few results have been reported on the ancient chordate selenoproteins.

**Results:**

A gene assembly algorithm SelGenAmic has been constructed and presented in this study for identifying selenoprotein genes from eukaryotic genomes. A method based on this algorithm was developed to build an optimal TGA-containing-ORF for each TGA in a genome, followed by protein similarity analysis through conserved sequence alignments to screen out selenoprotein genes form these ORFs. This method improved the sensitivity of detecting selenoproteins from a genome due to the design that all TGAs in the genome were investigated for its possibility of decoding as a Sec residue. Using this method, eighteen selenoprotein genes were identified from the genome of *Ciona intestinalis*, leading to its member of selenoproteome up to 19. Among them a selenoprotein W gene was found to have two SECIS elements in the 3'-untranslated region. Additionally, the disulfide bond formation protein A (DsbA) was firstly identified as a selenoprotein in the ancient chordates of *Ciona intestinalis, Ciona savignyi *and *Branchiostoma floridae*, while selenoprotein DsbAs had only been found in bacteria and green algae before.

**Conclusion:**

The method based on SelGenAmic algorithm is capable of identifying eukaryotic selenoprotein genes from their genomes. Application of this method to *Ciona intestinalis *proves its successes in finding Sec-decoding TGA from large-scale eukaryotic genome sequences, which fills the gap in our knowledge on the ancient chordate selenoproteins.

## Background

Selenium (Se), an essential trace element *in vivo*, is closely linked to Keshan disease, Kaschin-Beck disease, cancer and virus propagation. It also plays important roles in cell growth, proliferation and aging. Selenium *in vivo *is primarily present in various selenoproteins, which generally function as antioxidants to maintain the balance of redox state. The active site of selenoprotein is selenocysteine (Sec or U), the 21st amino acid encoded by a TGA codon in the open reading frame (ORF) of the gene [1]. Traditionally, TGA codon only signals the termination of protein synthesis; however, it can also be translated into a Sec residue when a specific stem-loop structure, designated as the Sec insertion sequence (SECIS) element, is located in the 3'-untranslated region (UTR) of a selenoprotein gene in eukaryotes and archaea, or located immediately downstream of the Sec-decoding TGA (designated as Sec-TGA) in bacteria [2-5]. The amino acid sequences flanking the active Sec residue are more conserved than other regions less functionally or structurally important in selenoproteins. These conserved regions play key roles in redox balance, metal combination, Sec-Sec/Sec-S bond formation and protein folding *in vivo*. Additionally, the Sec residue is highly analogous to the Cys in biochemical properties, which accounts for the fact that in most homologs of a selenoprotein the active Sec is replaced by Cys residues.

Several methods for *in silico *prediction of selenoprotein genes have been developed over the past decade, based on the structural characteristics of selenoprotein genes. These methods have been used separately or together for the identification of selenoproteins from the genomes or expressed sequence tag (EST) libraries in many species, such as human, fish, insects, green algae, nematodes and protozoa [6-11]. Recent application on the analyses of environmental metagenomic sequences have also been succeeded in finding prokaryotic selenoprotein genes [12,13]. To identify selenoprotein genes from the genome, several computational methods have to be combined for use, including the methods of RNA secondary-structure prediction, eukaryotic or prokaryotic ORF prediction, sequence alignment analysis and phylogenetic analysis. With the development and application of those methods, the size of selenoprotein family is growing, for example, the members of human selenoproteome increased from 14 to 25. In addition, up to 58 selenoprotein families have been identified recently in the Global Ocean Sampling (GOS) Project, which shed light on the evolution of selenoproteins according to their distribution in different species and environments.

**Figure 1:**
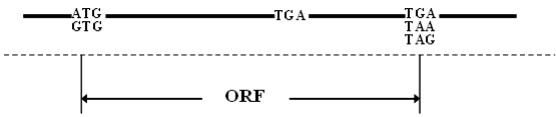
**A buildup of theoretical i-ORF containing a TGA codon in prokaryotic genomes**.

For the prediction of selenoprotein genes, it is indispensable to construct complete or partial ORFs containing the Sec-TGA codons. In a non-intron DNA sequence like prokaryotic genome, it is relatively easy to build a theoretical ORF containing a TGA codon termed "interrupted ORF" (i-ORF) as shown in Figure 1. However, in eukaryotic genome, the intron-exon structure of gene makes it difficult to build an i-ORF. Most of earlier studies on eukaryotic selenoprotein identification were performed by the following scheme. Firstly, RNA prediction algorithms were used to predict SECIS elements. Secondly, the SECIS elements were used to inform gene prediction algorithms to predict i-ORFs, of which a suitable SECIS element must be downstream [14]. The disadvantage of this scheme is that if any special-structure SECIS elements, which has not been discovered so far and included into the known SECIS models, existed in a newly sequenced genome, then no SECIS information can be used to inform the gene prediction algorithms to find the upstream ORFs. Naturally thinking, to identify the selenoproteins with special-structure SECIS, it must be able to predict i-ORFs without the help of SECIS information.

For eukaryotic genome, a SECIS-independent gene prediction approach was previously introduced in 2004 [11]. A modified gene prediction program named geneid was developed to identify 20 human selenoprotein genes. The gene assembly algorithm GenAmic used by geneid only builds the optimal gene structures with the TGA-containing exons having higher coding potential scores. Selenoproteins with lower score TGA-containing exons, such as human selenoproteins K, S and T, were not identified by the GenAmic based method. Although the sensitivity of this SECIS-independent gene prediction approach is high (20/25), it is not so good as the SECIS-dependent approach that discovered all 25 human selenoproteins [14]. In this paper, a new gene assembly algorithm named SelGenAmic was constructed to develop a similar SECIS-independent gene prediction method for the identification of selenoproteins. Compared with the GenAmic algorithm used by geneid, the SelGenAmic is more sensitive because its target is to build an optimal gene structure for each TGA. Thus no TGA codon is neglected for building i-ORFs. Finally, amino acid conservation assessment is used to find the real selenoproteins from these i-ORFs.

While much research has been done on the identification of selenoproteins of lower bacteria, unicellular eukaryotes, insects and higher vertebrates, only a few results have been reported for the sea squirts, one of the closest relatives in between invertebrates and vertebrates. Sea squirts are widely used for biological research in post-genomic era [15]. Abundant sequence data from the sea squirt genome project enable us to investigate selenoprotein distribution in these species, which provides valuable insights into selenium utilization and selenoprotein gene evolution [16,17]. Additionally, the sea squirt's worldwide distribution, short life cycle, and even its transparent body make it a potentially appropriate model organism for studying eukaryotic selenoproteins. Up to the present, only a few selenoproteins were found in the *Ciona intestinalis*, such as selenoprotein L (SelL), which was identified from EST sequences [18].

In this study, eighteen selenoproteins of *Ciona intestinalis *were identified from the genome by the method we developed according to the SelGenAmic algorithm. Combined with the SelL previously found from EST sequences, the members of selenoproteome in *Ciona intestinalis *increased to 19. Among those selenoproteins, disulfide bond formation protein A (DsbA) was the first time to be found as a selenoprotein in multicellular organisms. Its homologous DsbA in amphioxus and *Ciona savignyi*, two other ancient animals, were also identified to be selenoproteins.

## Results and Discussion

### Basic idea

TGA coding for Sec is one of the key characters of selenoprotein genes. If we scan all TGA codons from a genome, all Sec codons will be included. It is relatively easy to build a theoretical i-ORF from a prokaryotic genome as shown in Figure 1. This task can be carried out by finding a start codon and a stop codon in the nucleotide sequences flanking any TGA codon. All of i-ORFs can be translated into amino acid sequences and compared with known proteins to find potential selenoprotein genes. This method has been reported to be used for the discovery of rare amino acids, selenocysteine and pyrrolysine, in prokaryotes [19].

However building eukaryotic i-ORFs is much more difficult than that of prokaryotes. Many potential gene prediction signals like start codon ATG, stop codons TGA/TAA/TAG, and splice sites AG/GT can be found in the sequences flanking any TGA in a eukaryotic genome. It is rather complicated to choose suitable signals to build exons, and consequently suitable exons to build i-ORFs during selenoprotein identification.

**Figure 2:**
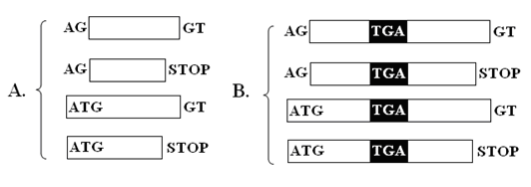
**Different types of exons used to assemble selenoprotein genes**. A. c-exons in which no in-frame TGA codon is allowed. B. i-exons in which an in-frame TGA codon is present and codes for a Sec residue. The signals such as splice sites AG and GT, start codon and stop codons are indicated in the figure.

**Figure 3:**
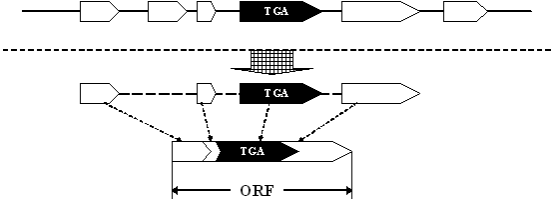
**Building an ORF containing a TGA codon**. The i-exon is indicated by a black arrow, and the c-exons by white arrows. An ORF containing an i-exon is formed by concatenating suitable c-exons and the i-exon.

To address this issue, a method was presented in this study. Firstly, all TGA codons were found from a genome, and supposed to be signals of Sec. All other signals such as start codon, stop codon and splice sites are also predicted. Secondly, common exons (c-exons) were built with common signals as shown in Figure 2A and interrupt exons (i-exons) containing TGA were built by concatenating common signals and TGA as shown in Figure 2B. Thirdly, the gene assembly algorithm SelGenAmic was used to build the best ORF for each i-exon. Figure 3 shows the process of building a best ORF for an i-exon. The best ORF which has the maximal coding potential is composed of this i-exon and other c-exons.

**Figure 4:**
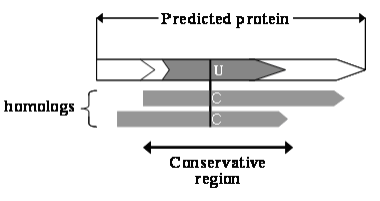
**A predicted protein which has conservative amino acid sequences flanking the Sec residue is more likely to be a selenoprotein**. In its homologs, the Sec (U) residue is expected to be replaced by Cys (C) residues.

If all i-exons and i-ORFs were enumerated from a genome to build a set, theoretically it should include all potential selenoprotein genes with Sec-TGA codons. However, the vast majority of these i-genes (genes containing Sec-TGA) will be biologically meaningless. To filter out such meaningless i-genes, the conservation of amino acid sequence in the local regions flanking the Sec residue (shown in Figure 4) was used to screen out i-genes that are more likely to be selenoprotein genes.

### General identification procedure

**Figure 5:**
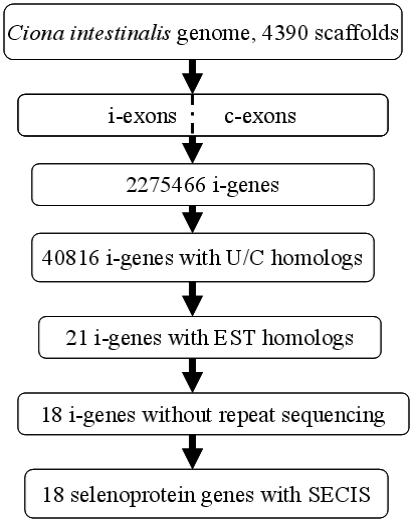
**The flow chart describing general procedures for the identification of selenoprotein genes from the *Ciona intestinalis *genome**.

General procedures of our method are shown in Figure 5 and described as follows.

(1) Obtaining i-exons and c-exons from the genome. The whole genome sequence of *Ciona intestinalis *containing 4390 scaffolds was scanned to find all TGA codons and other signals including ATG, TAA\TAG, and AG\GT. All i-exons and c-exons were built from these signals. The coding potential of any exon was calculated as the sum of the scores of the signals, plus the log-likelihood ratio of a Markov model for coding DNA.

(2) Assembling i-genes from i-exons and c-exons. For each i-exon, a best ORF which has the maximal coding potential score was built with our gene assembly algorithm SelGenAmic. 2275466 i-genes were built from *Ciona intestinalis*.

(3) Searching for Sec/Cys pairing and the conservation of its flanking regions. All i-genes were translated into amino acid sequences. Local sequences flanking the Sec residue were extracted for detecting similarity in the NCBI non-redundant (nr) protein database by the BLASTp program in order to obtain multiple sequence alignments. Those sequences were screened out with conservation in the local regions flanking the Sec residue, and alignments containing Sec/Cys pairing (simplified as U/C pair), i.e., the Sec-containing local sequence must have its homologous sequences contain Cys residues in the position of Sec in multiple alignments. 40816 i-genes were screened out by this step.

(4) Searching against EST databases and splicing the ESTs. Similarity analysis was performed against EST databases to obtain spliced ESTs for the 40816 i-genes. The local DNA sequences flanking the TGA of each i-gene were searched by BLASTn against the EST database of *Ciona intestinalis*. 21 i-genes were screened out after discarding the i-genes in which TGAs were analyzed to be the products of sequencing errors. Among those 21 i-genes, three of them are almost the same as another i-gene when comparing them with each other. Their corresponding genome segments are also very similar. Therefore, we concluded that the three analogous i-genes were caused by repeated sequencing and should be discarded. Finally, 18 i-genes were found to have complete ORFs and UTRs.

(5) Checking for SECIS elements. All 18 i-genes were found to have downstream SECIS elements in their UTRs, which further confirmed them as selenoprotein genes.

### Selenoproteins identified

The selenoprotein genes identified in this article are shown in Table 1. All of them are members of known selenoprotein families. The gene structures of selenoproteins in *Ciona intestinalis *are shown in the Supplemental Figure S1, S2 in the Additional file 1, along with the posi tions of exons, introns, UTRs and Sec-TGA codon. The secondary structures of SECIS elements and multiple sequence alignments of the conserved amino acid sequences flanking the Sec residue are shown in Supplemental Figure S3 and S4 in the Additional file 1

**Table 1:** Selenoproteins identified from the genome of *Ciona intestinalis*

Selenoprotein gene	Position
*selenoprotein N (SelN)*	2q
*Gpx like protein a (Gpx a)*	12p
*Gpx like protein b (Gpx b)*	1q
*Gpx like protein c (Gpx c)*	3q
*Gpx like protein c (Gpx d)*	14q
*Gpx like protein c (Gpx e)*	scaffold_161
*selenophosphate synthetase (SPS)*	8q
*selenoprotein W, 1 (SelW1)*	scaffold_63
*selenoprotein W, 2 (SelW2)*	1p
*15 kDa selenoprotein (Sel 15)*	scaffold_127
*iodothyronine deiodinase type 3 (DI 3)*	9p
*selenoprotein H (SelH)*	4q
*selenoprotein S (SelS)*	4q
*selenoprotein K (SelK)*	7q
*thioredoxin reductase (TR)*	8q
*selenoprotein O (SelO)*	6q
*selenoprotein T (SelT)*	3q
*DSBA like protein*	3q

* The genomic chromosome or scaffold from which the selenoprotein gene was identified. The abbreviated name of each selenoprotein is shown in parentheses.

Eight selenoproteins were found from the genome which was misannotated previously in the NCBI database. Brief information about these misannotated proteins is listed in Table 2, and comparisons between the gene structures of misannotated and newly identified genes are shown in Supplemental Figure S1 in the Additional file 1. No information was found in the NCBI database for the other newly identified selenoprotein genes in this article.

**Table 2:** The misannotated selenoprotein genes of *Ciona intestinalis*

Selenoprotein gene	Brief information in the NCBI database.
*SPS*	XM_002129490 PREDICTED: similar to selenophosphate synthetase 1 (LOC100182599)
*SelH*	XM_002124882 PREDICTED: similar to Selenoprotein H (SelH) (LOC100184326)
*SelO*	XM_002124815 PREDICTED: similar to predicted protein (LOC100179326)
*SelS*	XM_002131362 PREDICTED: similar to selenoprotein S (LOC100186840)
*DsbA*	XM_002126620 PREDICTED: hypothetical protein LOC100182954 (LOC100182954)
*SelK*	XM_002128679 PREDICTED: similar to selenoprotein K (LOC100184686)
*TR3*	XM_002131964 PREDICTED: similar to thioredoxin reductase 3 (LOC100178436)
*Gpx c*	XM_002121522 PREDICTED: Ciona intestinalis similar to C11E4.1 (LOC100182197)

It should be mentioned that *Ciona intestinalis SelL*, a selenoprotein gene found previously by Gladyshev, was not identified by our method. *SelL *gene was constructed from EST sequences, and proved to be a real selenoprotein of sea squirt before [18]. Comparing the sequences of *SelL *with the genome of *Ciona intestinalis *by Sim4 and BLASTn, no significantly similar regions of *SelL *were found in the genome data. The results implied that incomplete sequencing of the genome may cause the omission of genomic regions containing the *SelL *gene. The method developed in this paper is used to predict selenoproteins from genomes, thus it is impossible to find the *Ciona intestinalis SelL *gene from the genome data that do not include this gene.

### Unique *SelW1 *with two SECIS elements

**Figure 6:**
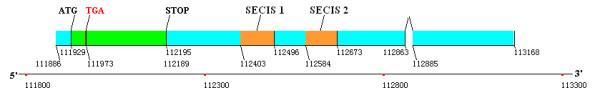
**Gene structure of *Ciona intestinalisSelW1***. The coding region is indicated by a green rectangle, the untranslated regions by blue rectangles, and the SECIS elements by orange rectangles. The intron is indicated by lines connecting the exons. The position of each site in the sequence of chromosome or scaffold is shown by numbers and the bottom coordinates.

Two SECIS elements were detected by SECISearch 2.19 in the 3'-UTR of the *SelW1 *gene of *Ciona intestinalis*. The gene structure is shown in Figure 6. All gene structures are schematically shown in this article as the 5'-end on the left. The complete ORF and two SECIS elements are located in the first exon. Two SECIS elements were named SECIS 1 and SECIS 2.

**Figure 7:**
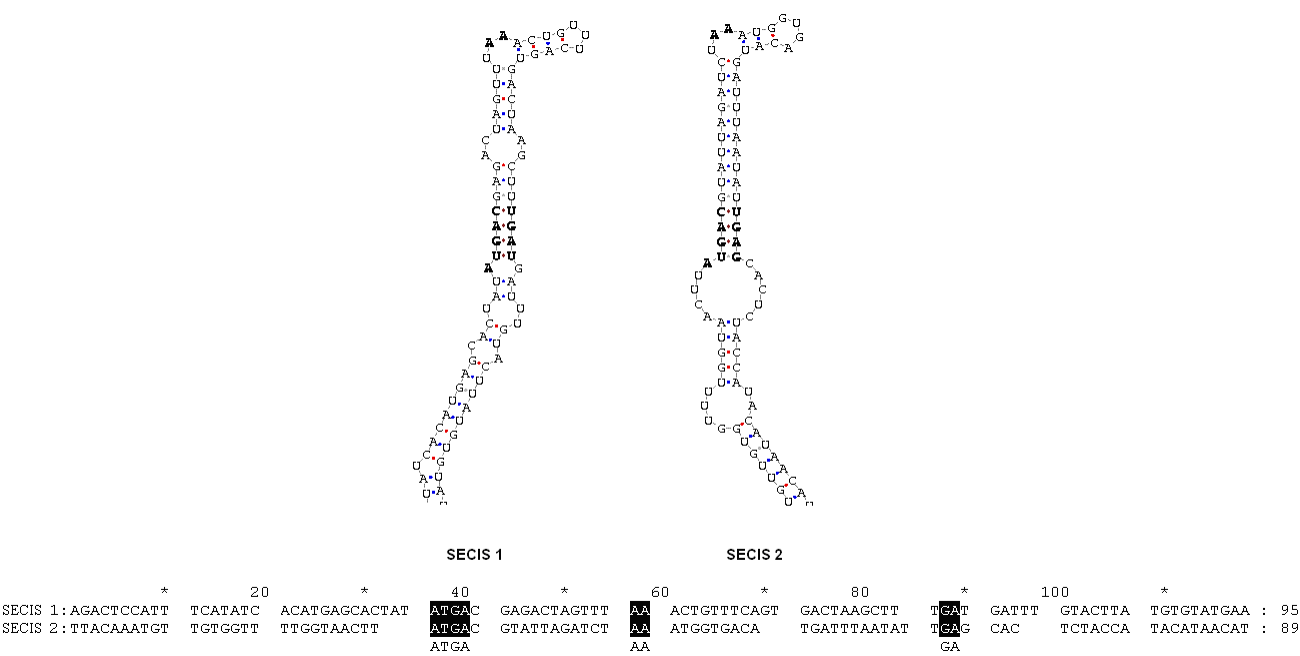
**Primary sequences and secondary structures of the two SECIS elements in the *Ciona intestinalis SelW1 *gene**. Core conserved functional sites are indicated by bold letters in the secondary structures and white letters on a black background in the primary sequences.

The primary sequences and secondary structures of the two SECIS elements are shown in Figure 7. Both of them belong to the form II SECIS element, which have an additional minihelix in the apical loop [2]. The two SECIS elements were both detected by SECISearch 2.19 under the "default" pattern to limit the conservation and energy cutoff. In addition, SECIS 2 could also be detected under the "strict" pattern. The COVE scores were 24.91 for SECIS 1 and 23.70 for SECIS 2, which are much higher than 15, the recommended COVE score cutoff for SECISearch2.19 [14]. The COVE score was calculated by matching the query sequence with the known SECIS secondary model. If the score exceeds 15, the query sequence is considered as a true SECIS according to the recommendation of SECISearch2.19. Therefore, both SECIS elements could be functionally active. Up to the present, two potential SECIS elements have only been found in *SelP *and human *Sep 15 *genes [6,20]. The *SelP *gene contains more than one Sec-TGAs, and experimental evidence has shown that its two SECIS elements are necessary for the efficient incorporation of multiple Sec residues into SelP. Whereas the *Sep 15 *gene contains only one Sec residue, and its upstream SECIS has been proved nonfunctional [21]. Interestingly, the predicted *Ciona SelW1 *gene also contains one Sec residue and two SECIS elements. Only SECIS 2 can be detected by the "strict" pattern of SECISearch 2.19. Those suggested that SECIS 2 has a higher possibility of being functional. From the characteristics of *Ciona SelW1 *gene structure, it seems that this gene is more analogous to human *Sep15 *that its SECIS 1 may be nonfunctional while SECIS 2 is used for Sec incorporation. However, conclusion can only be drawn after experimental results on functional analyses of these two SECIS elements.

### Selenoprotein *DsbA *found in multicellular organisms

**Figure 8:**
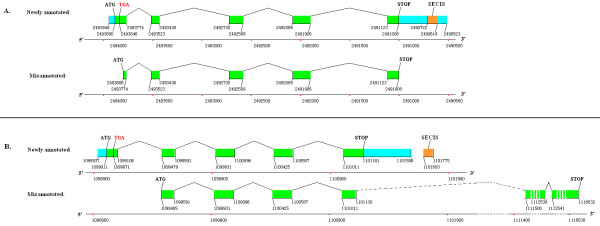
**Comparison between the gene structures of newly identified *DsbA *selenoprotein genes and the coding regions of originally misannotated genes**. **A**. *Ciona intestinalis DsbA*; **B**. *Branchiostoma floridae DsbA*. In the misannotated version, the downstream 2 exons and 1 intron are very long that they are indicated by broken line and interrupted rectangles.

Selenoprotein DsbA has only been found in multiple prokaryote species, microbial marine communities, symbiotic bacterium of a gutless worm, and *picoeukaryote Micromonas *[11,12,19,22-24]. In this paper, DsbA was firstly reported as selenoprotein in a multicellular organism, *Ciona intestinalis*. The structural information for selenoprotein DsbA gene of *Ciona intestinalis *is shown in Figure 8A indicated as newly annotated, while the originally released gene is indicated as misannotated, where the upstream part of the first exon, including the Sec-TGA, was misannotated as the UTR of the gene.

Interestingly, a selenoprotein *DsbA *gene was also identified in *Branchiostoma floridae *which was misannotated as hypothetical protein BRAFLDRAFT_120127 (gi|219424997|ref|XP_002210295.1|) in the present database [25]. The upstream sequence of the coding region in this amphioxus gene was extracted from its mRNA, and translated into an amino acid sequence where the original start codon, ATG, was decoded as a methionine (Met, or M) and a TGA decoded as a Sec. The translated sequence was analysed by BLASTp against the NCBI nr database. The complete ORF of the selenoprotein *DsbA *gene was constructed by extending its aligned ESTs of *Branchiostoma floridae*. However, not enough ESTs were found to be extended to the UTR for SECIS search. Thus, the ORF downstream sequence was extracted from the genome to search for SECIS element. A positive hit was found by SECISearch 2.19. The gene structure of this newly identified selenoprotein *DsbA *is shown in Figure 8B indicated as newly annotated, while the originally annotated coding region in the NCBI database is indicated as misannotated. The first exon of the new gene containing the Sec-TGA codon was misannotated as the 5'-UTR in the original version, and the last exon of the new gene including the stop codon and 3'-UTR was missing originally. Two long exons and a long intron were found at the end of the original gene. No similar EST sequence or amino acid sequence has been released for these two exons.

**Figure 9:**
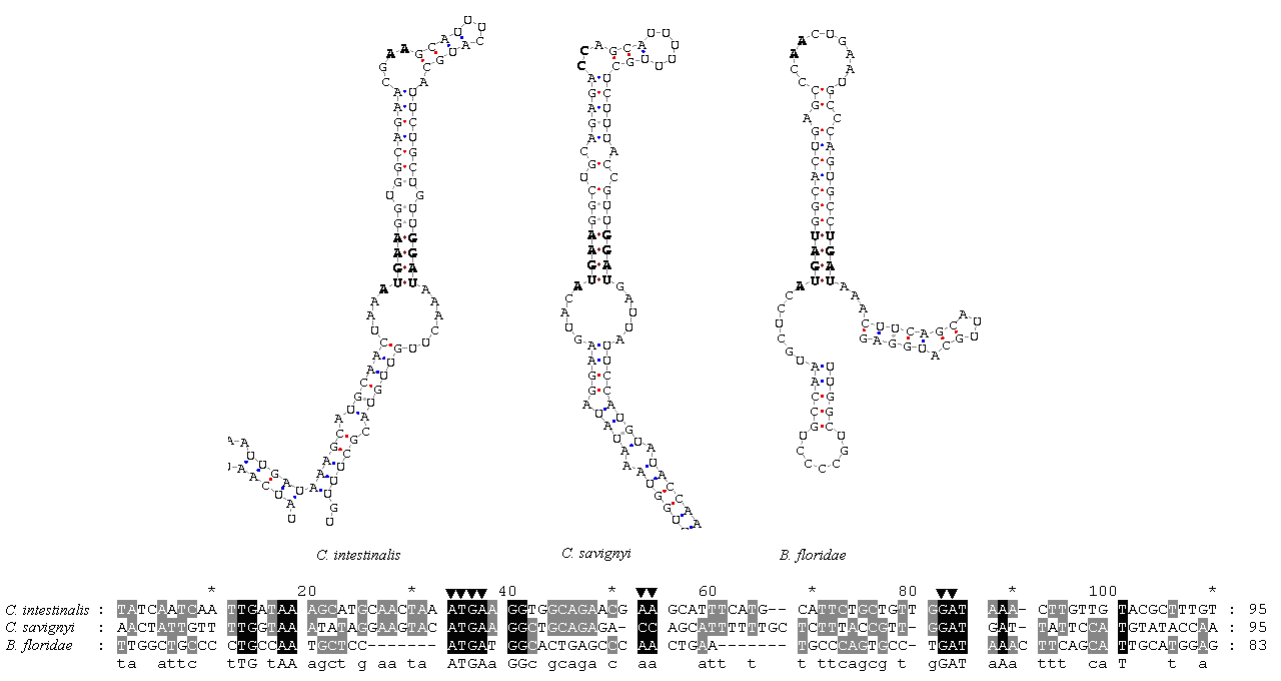
**Primary sequences and secondary structures of the SECIS elements of *Ciona intestinalis, Ciona savigyni *and *Branchiostoma floridae DsbA *selenoprotein genes**. Conserved functional sites are indicated by ▼.

The primary sequences and secondary structures of the SECIS elements in the newly identified *DsbA *selenoprotein genes from three species are shown in Figure 9. Homologous analysis by multiple alignments of SECIS sequences in Figure 9 shows that the two SECIS elements from the *Ciona DsbA *selenoprotein genes are more similar than the one from *Branchiostoma floridae*. In addition, both *Ciona *SECIS elements belong to the form II SECIS that has an additional minihelix in the apical loop. The COVE scores of the SECIS elements of *Ciona intestinalis*, *Ciona savigyni *and *Branchiostoma floridae *were 22.40, 17.89 and 18.83, respectively. An unpaired CC motif is found in the apical loop of *Ciona savigyni *SECIS element as a conserved site, while the AA motif was found in *Ciona intestinalis *and *Branchiostoma floridae *SECIS elements. The AA motif of a SECIS element has been reported in most selenoprotein genes of different organisms, while the rare CC motif has only been reported in human, rat and mouse selenoprotein M [26].

The thiol/disulfide interchange protein DsbA, a member of the thioredoxin family of disulfide oxidoreductases, catalyzes disulfide bond formation by donating its disulfide to newly translocated proteins in many prokaryotes [27]. Eukaryotic protein disulfide isomerase (PDI) has a similar function, and both proteins have similar redox active levels [28]. DsbA contains a redox-active CXXC motif imbedded in a TRX fold. The active CXXC motifs can be found in many other selenoproteins. Research on the structure and function of the CXXC motif in *Escherichia coli *DsbA has shown that the upstream Cys is exposed on the surface of protein, and the downstream Cys is embedded in the 3-D structure [29]. The upstream Cys has a very low pK_a _value (≈3.5), and is completely ionized under physiological pH conditions [30]. Mutational analyses have revealed that the upstream Cys residue can catalyze thiol/disulfide interchange reaction without the presence of the downstream Cys [31]. These studies suggest that the upstream Cys is more important than the downstream Cys of DsbA. Interestingly, the upstream Cys is replaced with Sec in our newly identified three DsbA selenoproteins. Due to the higher redox activity of Sec compared with that of Cys, it is reasonable for us to deduce that selenoprotein DsbAs with Sec in place of Cys in the reactive centres are more functionally active than non-selenoprotein DsbAs.

### Evolutionary analyses of DsbA

**Figure 10:**
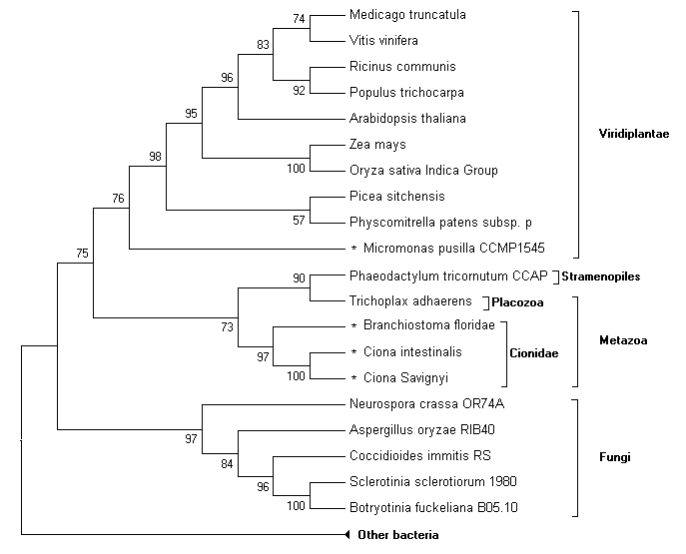
**Phylogenetic tree of eukaryotic DsbA**. Selenoproteins are marked by an asterisk. Bootstrap values are shown at each branchpoint to indicate the reliability of this tree.

**Figure 11:**
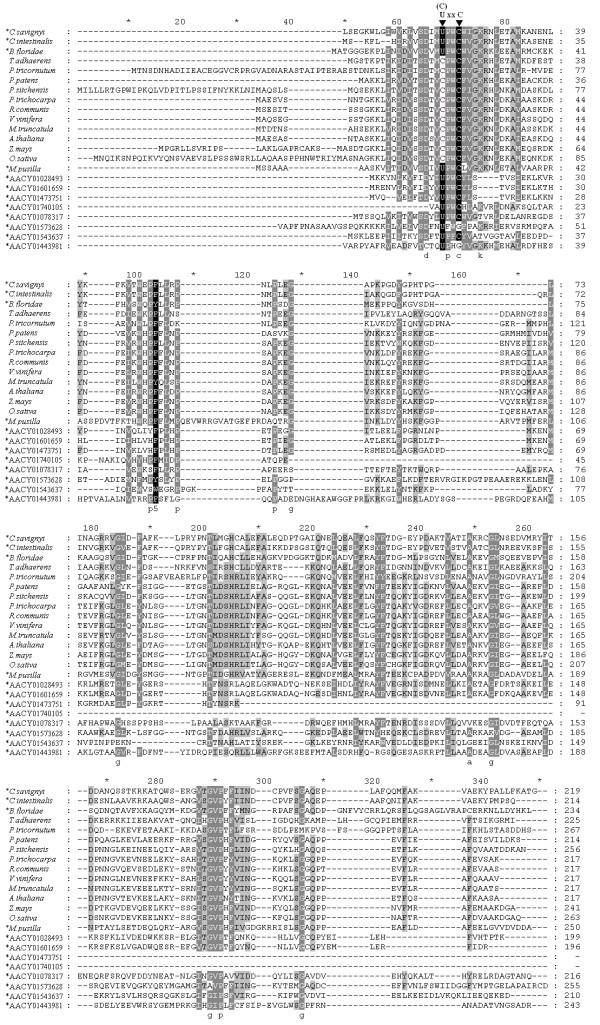
**Multiple alignments of eukaryotic DsbA-like proteins and several prokaryotic DsbA-like selenoproteins from the microbial selenoproteome of the Sargasso Sea**. Sec and Cys residues in the active site U(or C)XXC are marked on top. Species names of eukaryotes and sequence names from the Sargasso Sea are listed on the left. Selenoproteins are marked by an asterisk.

The 100 most similar protein sequences to DsbA were obtained from the NCBI nr database by BLASTp program. Sequences from the same species were deleted, leaving 67 homologs of DsbA, including the newly identified DsbA selenoproteins. Those DsbA homologs were aligned and phylogenetically analysed. Most of the DsbAs (47 of 67) are bacterial proteins, and the remaining 20 are eukaryotic. A phylogenetic tree of these 20 eukaryotic DsbA proteins is shown in Figure 10. The multiple sequence alignments of these 20 proteins, along with 8 prokaryotic DsbA selenoproteins found previously in the Sargasso Sea microbial selenoproteome, are shown in Figure 11. The putative CXXC active sites and Sec residues are highlighted.

Phylogenetic analysis shows that eukaryotic DsbA proteins can be clearly classified into three groups: the fungi, viridiplantae and metazoa. No selenoprotein DsbA was found in the fungi group. Only one selenoprotein DsbA was found in the viridiplantae group, which belongs to *Micromonas pusilla*, a unicellular plant. The three newly predicted DsbA selenoproteins belong to the metazoa group, cionidae subgroup. They are most closely related to each other. According to the phylogenetic analysis, DsbA is widely distributed in prokaryotes. The utilization of selenium to form Sec in DsbA diverged during the evolution from prokaryotes to eukaryotes. Since both selenoprotein DsbA and non-selenoprotein DsbA (the Cys-containing form) could be found in prokaryotes, metazoa, and viridiplantae (see Figure 10), a hypothesis was deduced that the evolution of selenoprotein DsbA occurred prior to the separation of animal and plant kingdoms. Additionally, the Sec residue was lost or replaced by Cys in land plants [8]. The hypothesis is supported by the evidence that only an early-diverging unicellular plant in the viridiplantae has a selenoprotein DsbA, while other land plants have the Cys-containing proteins.

Sea squirts and amphioxus are important transitional species, bridging the gap between vertebrates and invertebrates. Amphioxus is even considered as the invertebrate that is most evolutionarily close to vertebrates. The placozoan *Trichoplax adhaerens*, a close relative to the three cionidae DsbA selenoproteins in the phylogenetic tree, is arguably the simplest free-living animal, and therefore may represent a primitive metazoan form [32]. Genome project research on these ancient species has provided important information for studying the evolution of vertebrates, and even metazoans. Interestingly, selenoprotein DsbAs were found in these primitive chordates, while only Cys-containing form DsbAs were found in the primitive metazoan. No DsbAs were found in vertebrates through homologous analysis. The reason could be that DsbA is possibly replaced during evolution by another enzyme with similar function, such as DPI or other proteins in the thioredoxin family. In higher vertebrates, the DsbA family was completely replaced by other proteins with similar function, leading to the disappearance of DsbA selenoproteins.

### Relative merit of the SelGenAmic-based method

The selenoprotein identification scheme presented herein (an 'i-ORF-assembly-first, Sec-homology-second, SECIS-search-third' approach) differs from the reported methods in some ways. The methods based on SECIS search required the finding of SECIS elements to inform their gene-prediction-algorithms the proper sequences for predicting i-genes [7]. However, it is possible for some new selenoprotein genes to have certain special SECIS structures that have not been discovered up to the present, thus can't be detected by available known programs. In this case, the SECIS-search based approach is impossible to find those special selenoprotein genes. Although the method based on read-through similarity analysis (RSA) had made up this shortage [33], it could only be applied to identify prokaryotic selenoprotein genes but not the eukaryotic due to their complicated intro-exon structures. In this sense, our method has the merit of independently finding possible i-ORFs for all TGA codons in eukaryotic genomes without the help of SECIS information. This is because the SelGenAmic algorithm could enumerate all i-ORFs for all TGA codons in a eukaryotic genome. Therefore, even with special SECIS structures, those selenoproteins will not be lost in the analysis using the SelGenAmic-based method. They can be found during protein sequence conservation analysis.

In fact, the SECIS-independent-prediction method was firstly introduced to find human and fugu selenoproteins in 2003 [11,14]. However, its ORF prediction step is different from ours. The SelGenAmic algorithm we developed in this paper is to assemble i-ORF for each TGA in genomic sequences, while the GenAmic algorithm reported previously is to assemble selenoprotein genes and standard genes at the same time, using stronger coding bias for the sequence downstream Sec-TGA codon to discriminate real Sec-TGA codons and others. Although it was performed with rather high sensitivity (80%), some selenoproteins, such as SelK, SelT and SelS, were still missing during human genome analysis. To overcome this shortage, the SelGenAmic algorithm was developed to maximize the sensitivity in such way that an optimal i-ORF was built for each TGA codon, without skipping any TGA codon even with low coding bias for the downstream sequence. The SelGenAmic-based method was also applied to human genome to detect its sensitivity, and it successfully identified the whole 25 human selenoproteins as shown as Supplemental Figure S5 in the Additional file 1. However, the improvement was achieved at the expense of dealing with a huge amount of data (more than 2 million biological meaningless i-genes in *Ciona*). These false positive predictions could be removed through protein conservation alignments.

Another merit of the SelGenAmic-based method is that it can find the alternative splicing genes of selenoproteins. Some selenoprotein genes, such as human DI2, have more than one alternative splicing forms. The Sec residues of two human DI2 forms were coded by different TGA codons in the gene. Because earlier methods, like Geneid_SP, only considered one of these two TGAs as the best Sec codon, they could not detect the alternative splicing form of DI2 containing another Sec-TGA codon. Using our method, both forms of human DI2 were identified (data not shown), so were the two forms of DI2 in the armadillo genome (shown as Supplemental Figures S6, S7 and S8 in Additional file 1).

## Conclusion

A eukaryotic selenoprotein identification method based on a gene assembly algorithm SelGenAmic was presented in this paper. It focuses on the prediction of ORF containing a Sec-TGA codon in the eukaryotic genomes. With the aid of this method, 18 selenoprotein genes were identified from the genome of *Ciona intestinalis*, leading to the member of its selenoproteome up to 19. Among them, a unique selenoprotein gene of *Ciona intestinalis SelW1 *contained two SECIS elements while others had only one SECIS element in their 3'-UTRs. In addition, DsbA was firstly found to be a selenoprotein in multicellular organisms, like *Ciona intestinalis, Ciona savignyi*, and *Branchiostoma floridae*. The existence of DsbA selenoproteins in multicelluar organisms provides important information for the exploration of the evolution of selenium utilization in invertebrates and vertebrates.

## Methods

### Data resources

The genome sequences were downloaded from the Ensembl Project Genome Databases http://www.ensembl.org. The release number of the genome data is JGI2.48 for *Ciona intestinalis*. The text file size of the genome data of *Ciona intestinalis *is about 172 Mb, containing approximately 176,000,000 bp, 4,390 scaffolds. All available *Ciona intestinalis *EST sequences (1,205,981 at the time of our analyses) were extracted from the NCBI database.

### Construction of ORFs containing Sec-TGAs

The program Geneid (version 1.2a) [34] was used to obtain all common gene signals, such as start codon, splice sites, stop codons, and common potential exons, from genomic sequences. A series of PERL programs were edited to obtain all TGA codons from a genome, and build i-exons from common signals and TGA codons. The coding potential was obtained by Geneid with specific parameter file of *...Ciona intestinalis *(downloaded from http://genome.crg.es/software/geneid/index.html). A PERL program was edited based on the i-gene assembly algorithm, SelGenAmic, to construct all i-genes from common exons and i-exons.

### Assembly algorithm SelGenAmic

The algorithm SelGenAmic is developed from GenAmic to solve the problem of finding an optimal ORF for each i-exon. The word optimal here means that the coding potential score of such ORF is bigger than any other ORFs composed of this i-exon and other suitable c-exons.

The input data of SelGenAmic are all i-exons and c-exons along with their information such as coding potential, position and coding frame. Let *E *= {*e*_1_, *e*_2_, ..., *e*_*k*_, (*k *≥ 0, *k *is an integer) be the set of exons. The coding potential of these exons are shown as *P*(*e*) for each *e *∈ *E *in this paper.

Let *C *= {*c*_1_, ..., *c*_*m*_, (*m *≥ 0, *m *is an integer) and *T *= {*t*_1_, ..., *t*_*n*_, (*n *≥ 0, *n *is an integer) be the sets of c-exons and i-exons. Obviously, *E *= *C*∪*T*.

The principle to constrain the algorithm to choose suitable exons for concatenation is described as a function *M*.

The function M describes the relation of two exons *e*_*a *_and *e*_*b*_(*e*_*a *_∈ *E*, *e*_*b *_∈ *E*), which can be concatenated legally in numerical order. The word legal here means *e*_*a *_and *e*_*b *_are frames compatible, non-overlapping, and adjoining splice sites matched.

Firstly we recall the concepts of gene assembly. A gene assembly *g *is a sequence consisting of exons *e*_1'_, ... *e*_*q' *_from *E *(*e*_*i' *_∈ *E*). Thus a legal gene assembly can be described as

*g *= <*e*_1'_, ... *e*_*q' *_>, where for all *e*_*i' *_in *g*, *M*(*e*_*i'*_, *e*_(*i*+1)'_) = 1.

The coding potential of a gene assembly is the sum of scores of assembled exons: *P*(*g*) = *P*(*e*_1'_)+...+*P*(*e*_*q'*_). The problem to find an optimal g could thus be interpreted as to search for the gene assembly *g *with maximum *P*(*g*), i.e., for all other genes *g' *constructed from *E*, *P*(*g*)> = *P*(*g'*)

Thus the target of SelGenAmic is that, for each *t*_*i *_∈ *T*, finding an optimal gene assembly *g*(*t*_*i*_) = <*c*_1'_, ..., *c*_*k'*_, *t*_*i*_, *c*_1"_, ..., *c*_*k"*_>, where *c*_*i' *_∈ *C*, *c*_*i" *_∈ *C*, *M*(*c*_*k'*_, *t*_*i*_) = 1, *M*(*t*_*i*_, *c*_1"_) = 1, *M*(*c*_*i'*_, *c*_(*i*+1)'_) = 1, *M*(*c*_*i"*_, *c*_(*i*+1)"_) = 1, i.e., for the set *T *= {*t*_1_, ..., *t*_*n*_, build a set of optimal gene assemblies {*g*(*t*_1_), ..., *g*(*t*_*n*_).

This problem is equivalent to find the best upstream assembly *g*_*u *_= <*c*_1'_, ... *c*_*k'*_, *t*_*i*_,> and the best downstream assembly *g*_*d *_= <*t*_*i*_, *c*_1"_, ... *c*_*k"*_> for each *t*_*i*_.

To solve such problem, we introduced six concepts. (1) the best upstream assembly(BUA); (2) the coding potential of BUA (CpBUA); (3) the best upstream adjoining exon (BUE); (4) the best downstream assembly (BDA); (5) the coding potential of BDA (CpBDA) and (6) the best downstream adjoining exon (BDE).

The concepts (1), (2) and (3), were used in the GenAmic algorithm of Geneid program to calculate the optimal assembly from common exons [35]. The concept (1) BUA was described as that for each exon *c*_*i *_from *C *it is possible to find a best assembly ended with *c*_*i*_, *i.e., g*_*u*_(*c*_*i*_) = <*c*_1'_, ... *c*_*i*_>, where for all other assembly *g' *ended with *c*_*i*_, *P*(*g*_*u*_(*c*_*i*_))> = *P*(*g'*). The coding potential *P*(*g*_*u*_(*c*_*i*_)) is the CpBUA (concept (3)) of *c*_*i*_.

The concept (2) BUE was described as that for each *c*_*i *_from *C*, it is possible to find a best upstream adjoining exon *c*_*j*_, if and only if

*1. M*(*c*_*j*_, *c*_*i*_) = 1, and

2. for all other *c' *∈ *C*, if *M*(*c'*, *c*_*i*_) = 1, *P*(*g*_*u*_(*c*_*j*_)) > = *P*(*g*_*u*_(*c'*)).

Here we use function *G *to describe the relationship between a c-exon *c*_*i *_and its BUE *c*_*j*_: *G*(*c*_*i*_) = *c*_*j*_.

Obviously, the BUA *g*_*u*_(*c*_*i*_) of *c*_*i*_, can be obtained by concatenating its BUE *c*_*i *_and the BUA *g*(*c*_*j*_) of *c*_*i*_:

So that, it can be easily concluded that if all the BUE for each *c *∈ *C *were known, the BUA can be easily calculated as follow:

*g*_*u*_(*c*_*i*_) = <*c*_0_, ..., *G *(*G*(*c*_*i*_)), *G*(*c*_*i*_), *c*_*i *_>, (*c*_0 _is used to describe the first exon of this assembly).

In Geneid, the author of GenAmic algorithm used dynamic programming to obtain all the BUE *G*(*c*) for every *c *∈ *C *[36]. Using the GenAmic, we can easily build 3 sets for *C *= {*c*_1_, *c*_2_, ..., *c*_*k*_:

the set of BUE *G*(*c*_1_), *G*(*c*_2_), ..., *G*(*c*_*k*_)},

the set of BUA { *g*_*u*_(*c*_1_), *g*_*u*_(*c*_2_), ..., *g*_*u*_(*c*_*k*_)}, and

the set of CpBUA *P*(*g*_*u*_(*c*_1_)), *P*(*g*_*u*_(*c*_2_)), ..., *P*(*g*_*u*_(*c*_*k*_)).

Knowing how to calculate the BUA *g*_*u*_(*c*_*i*_) for each c-exon *c*_*i *_∈ *C*, we can use the same method to build the BUA *g*_*u*_(*t*_*i*_) for each i-exon *t*_*i *_∈ *T*:

*g*_*u*_(*t*_*i*_) = <*c*_1'_, ... *c*_*k'*_, *t*_*i *_> = <*g*_*u *_(*G*(*t*_*i*_)), *t*_*i *_> = <*g*_*u *_(*c*_*u*_), *t*_*i *_>, where *c*_*u *_is the BUE of *t*_*i*_.

The BUE *c*_*u *_∈ *C *is a c-exon, and the set of BUA { *g*_*u*_(*c*_1_), *g*_*u*_(*c*_2_), ..., *g*_*u*_(*c*_*k*_)} for each *c *∈ *C *can be obtained with GenAmic algorithm in linear time. Thus, if all the BUE *c*_*u *_for each i-exon *t*_*i *_∈ *T *can be obtained, its BUA can also be produced.

The BUE G(*t*_*i*_) = *c*_*u *_can be found in the following way. We find all exons *c' *satisfying *M*(*c'*, *t*_*i*_) = 1, and their CpBUA from {*P*(*g*_*u*_(*c*_1_)), *P*(*g*_*u*_(*c*_2_)), ..., *P*(*g*_*u*_(*c*_*k*_)). By comparing their coding potentials, the *c*_*u *_with maximal *P*(*g*_*u*_(*c*)) can be found.

**Figure 12:**
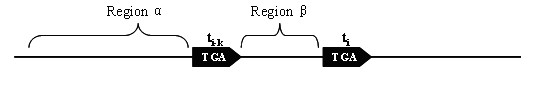
**The regions in which c-exons are used to search for the best upstream adjoining exon of *t*_*i*_**.

As shown in Figure 12, all c-exons *c" *∈ *C *located upstream of *t*_*i *_and satisfying *M*(*c", t*_*i*_) will be searched for the *c*_*u *_of *t*_*i*_. An i-exon *t*_*i*-*k *_can be found to divide the genome into 2 regions. The *t*_*i*-*k *_is a exon for which all c-exons *c" *satisfying *M*(*c", t*_*i*-*k*_) = 1 are also satisfying *M*(*c", t*_*i*_) = 1. Obviously, the c-exon with maximal CpBUA in region *α *is the BUE of *t*_*i*-*k*_. Then only *c' *in region *β *satisfying *M*(*c'*, *t*_*i*_) = 1 will be searched for the c-exon with maximal CpBUA. Let *A *and *B *be the sets of c-exons in the region *α *and *β*, respectively, then to find the *c*_*u *_for *t*_*i*_, can be described as follow:

With this equation, all BUE(s) of *t*_*i *_∈ *T *can be calculated recursively with one scan of the genome in linear time, due to the reason that only the region *β *for each *t*_*i *_is needed to be searched.

Then the set of BUE for *T *is obtained as *G*(*t*_1_), *G*(*t*_2_), ..., *G*(*t*_*k*_)} and

the set of BUA for *T *is obtained as *g*_*u*_(*G*(*t*_1_)), *g*_*u*_(*G*(*t*_2_)), ..., *g*_*u*_(*G*(*t*_*k*_)).

The BDA, BDE and CpBDA for all *c *∈ *C *can be obtained in similar way. Let *g*_*d*_(*c*_*i*_) be the BDA of *c*_*i*_, and let function *G*^*r *^describe the relation of *c*_*i *_and its BDE *c*_*j*_:

*G*^*r*^(*c*_*i*_) = *c*_*j*_, if and only if

1. *M*(*c*_*i*_, *c*_*j*_) = 1 and

2. for all other *c' *∈ *C*, if *M*(*c*_*i*_, *c'*) = 1, then *P*(*g*_*d*_(*c*_*j*_))> = *P*(*g*_*d*_(*c'*)).

Thus the set of BDA, BDE and CpBDA for all *c *∈ *C *can be produced similarly as follows:

*G*^*r*^(*c*_1_), *G*^*r*^(*c*_2_), ..., *G*^*r*^(*c*_*k*_)},

{ *g*_*d*_(*c*_1_), *g*_*d*_(*c*_2_), ..., *g*_*d*_(*c*_*k*_)}, and

*P*(*g*_*d*_(*c*_1_)), *P*(*g*_*d*_(*c*_2_)), ..., *P*(*g*_*d*_(*c*_*k*_))}.

Then the set of BDA for *T *is obtained as *g*_*d*_(*G*^*r*^(*t*_1_)), *g*_*d*_(*G*^*r*^(*t*_2_)), ..., *g*_*d*_(*G*^*r*^(*t*_*k*_)).

By concatenating BUA and BDA, the best assembly for *t*_*i *_is constructed as *g *= <*g*_*u*_(*G*(*t*_*i*_)), *t*_*i*_, *g*_*d*_(*G*^*r*^(*t*_*i*_)) >.

Thus, for each i-exon an optimal assembly can be constructed in linear time.

### Homology analysis

The NCBI nr protein database was downloaded from the NCBI ftp server in June of 2008, containing 6,598,440 protein sequences, 2,257,741,895 total letters. BLAST programs (version 2.2.18) [36] were also obtained from the NCBI ftp server at ftp://ftp.ncbi.nih.gov/blast/db/. All i-genes were searched by the program BLASTp with an E-value cutoff at 1. All similar sequences detected were used to create multiple sequence alignments with ClustalW (version 1.83) [37]. The conservative motif containing the Sec residue of an i-gene was analysed by the program using a motif search algorithm like MAME.

### Gene structure analysis

EST sequences were downloaded and compared with all predicted selenoprotein genes using the program BLASTn. Highly similar EST sequences were spliced by SeqMan program from the DNASTAR package http://www.dnastar.com/ and analysed for the selenoprotein gene structure. The constructed genes were homologously compared to genomic sequences with the program Sim4 [38] to find the locations of exons and introns in the genome, shown as position numbers in Figure 3, Figure 5 and Supplemental Figure S1, Figure S2 in the Additional file 1.

### Search for SECIS elements

RNAfold (version 1.7.2) [39] and PatScan [40] were automatically used by a PERL program to detect SECIS-like structures from genome sequences. The SECIS patterns used in the present paper are the same as that in the search of human SECIS [14]. These patterns together with the SECISearch program in PERL language were kindly provided online by Doctor Charles (Karolos) Chapple http://genome.imim.es/~cchapple/. The COVE score of SECIS-like structures were evaluated by the online program SECISearch (version 2.19) [14].

### Phylogenetic analysis

Multiple alignments of amino acid sequences were generated using the ClustalX program (version 1.83) [41]. The unrooted phylogenetic tree with unscaled distance branches were generated using the program MEGA3.1 http://meme.sdsc.edu/meme4_1/intro.html with the Neighbor-Joining method. Tests of the phylogenic analyses were done by 1000 replications of the Bootstrap algorithm.

## Authors' contributions

LJ carried out the whole research work including program edit, algorithm design, selenoprotein identification from the genomes, and draft making. QL was responsible for the project design, key-issue discussion and manuscript writing. JN was responsible for the project design, progress and coordination. All authors read and approved the final manuscript.

## Supplementary Material

Additional file 1 **Additional information of newly predicted selenoproteins**. The following additional data are included within the additional file1. Gene structures of the newly identified selenoprotein genes of *Ciona intestinalis*, and comparison between the newly identified version and misannotated version of these genes are shown in Supplemental Figure S1 and S2. The secondary structures of the SECIS elements of *Ciona intestinalis *selenoprotein genes are shown in Supplemental Figure S3. Multiple alignments of all newly identified selenoproteins and their homologous sequences are shown in Supplemental Figure S4. Multiple alignments of all human selenoproteins predicted by the SelGenAmic-based method and their homologous sequences are shown in Supplemental Figure S5. Information on chromosome number and ORF position of these genes were also shown in Supplemental Figure S5. Gene structures of the two alternative splicing forms of DI2 predicted from the armadillo genome were shown in Supplemental Figure S6, along with their multiple alignments (Supplemental Figure S7) and secondary structure of SECIS elements (Supplemental Figure S8). Click here for file
