# Supplementary material for: In silico identification of the sea squirt selenoproteome
Source: BMC Genomics. 2010 May 10;11:289. doi: 10.1186/1471-2164-11-289 (PMC2874816; doi:10.1186/1471-2164-11-289)
Supplement: Additional file 1 — Additional information of newly predicted selenoproteins. The following additional data are included within the additional file1. Gene structures of the newly identified selenoprotein genes of Ciona intestinalis, and comparison between the newly identified version and misannotated version of these genes are shown in Supplemental Figure S1 and S2. The secondary structures of the SECIS elements of Ciona intestinalis selenoprotein genes are shown in Supplemental Figure S3. Multiple alignments of all newly identified selenoproteins and their homologous sequences are shown in Supplemental Figure S4. Multiple alignments of all human selenoproteins predicted by the SelGenAmic-based method and their homologous sequences are shown in Supplemental Figure S5. Information on chromosome number and ORF position of these genes were also shown in Supplemental Figure S5. Gene structures of the two alternative splicing forms of DI2 predicted from the armadillo genome were shown in Supplemental Figure S6, along with their multiple alignments (Supplemental Figure S7) and secondary structure of SECIS elements (Supplemental Figure S8). [file 1471-2164-11-289-S1.DOC]

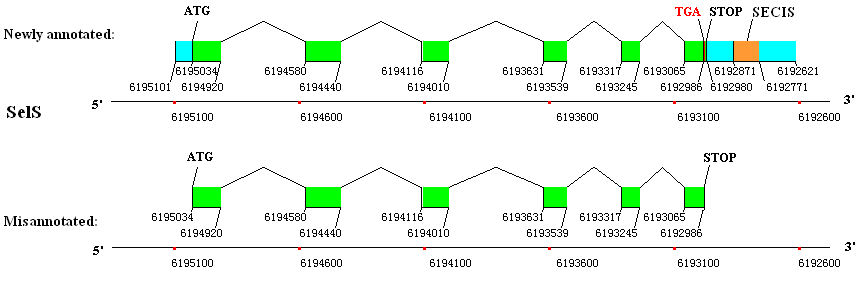


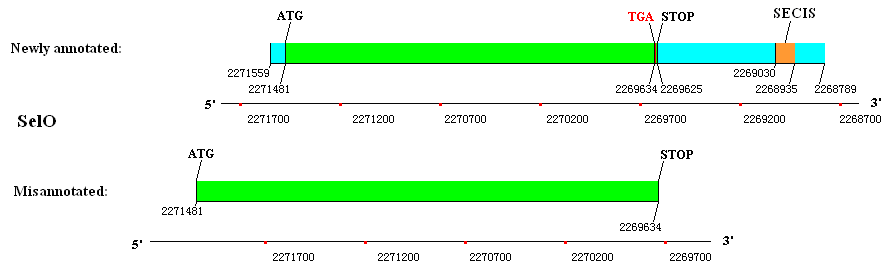


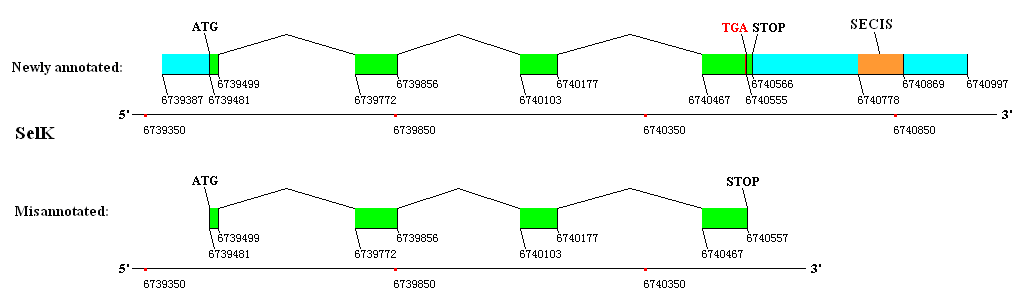


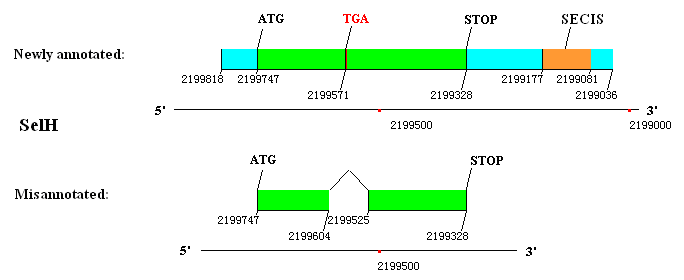


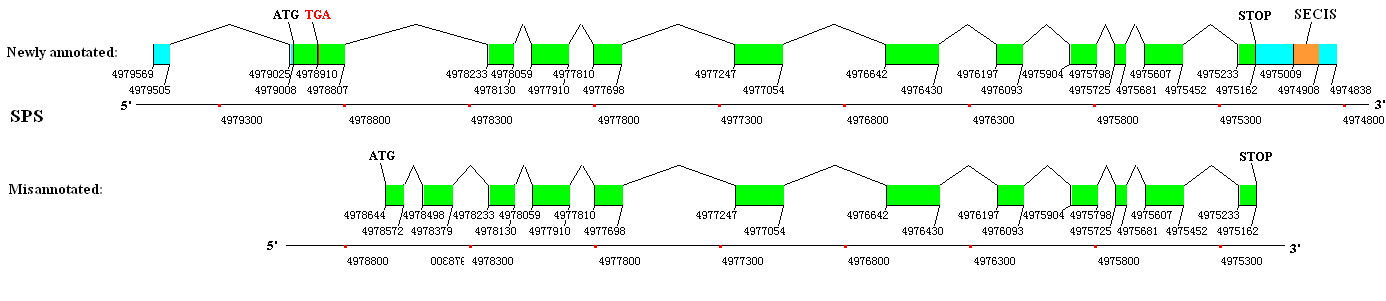


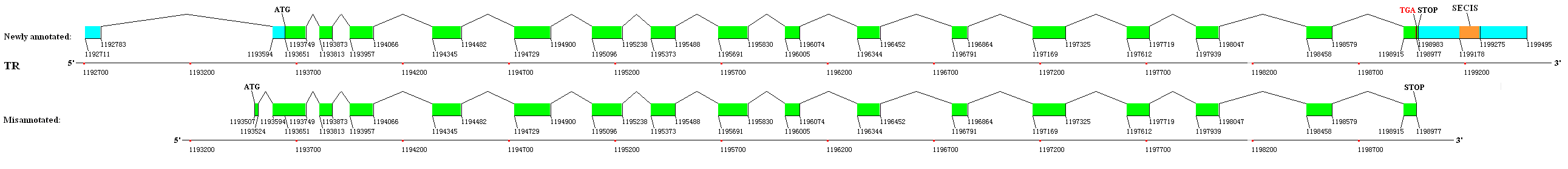


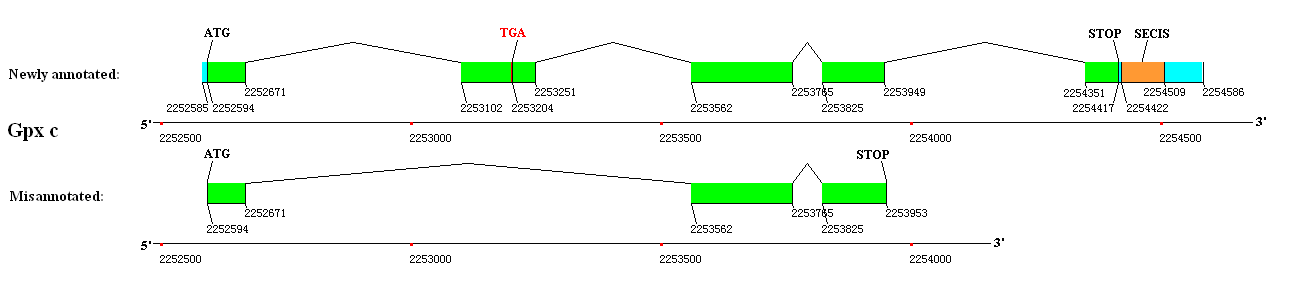


## Figure S1. Comparison between the newly identified *Ciona intestinalis* selenoproteins, including SelS, SelO, SelK, SelH, SPS, TR and Gpx c, and their originally misannotated versions.


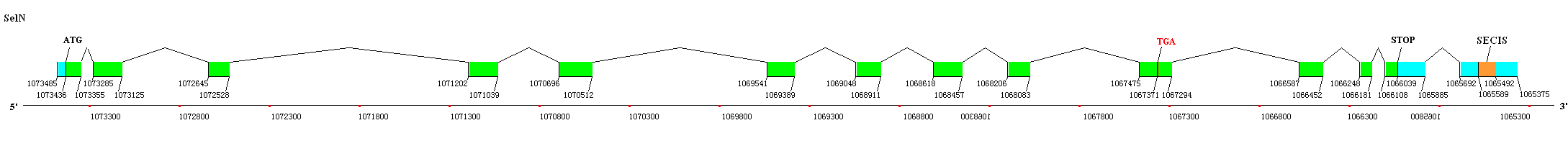


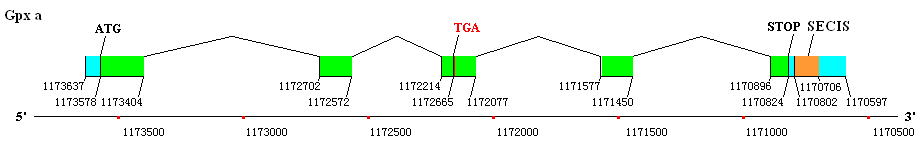


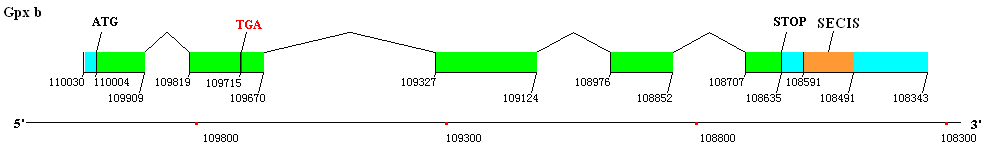


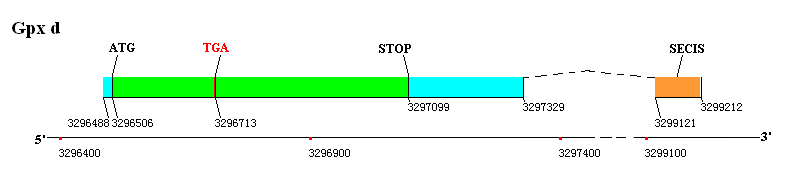


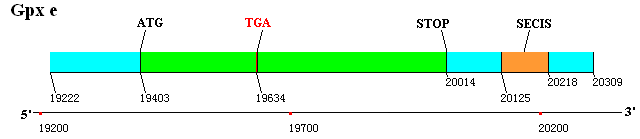


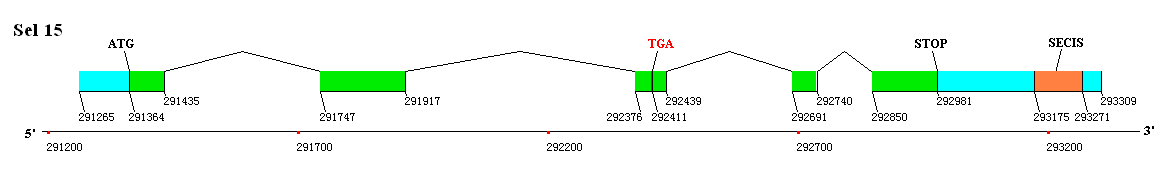


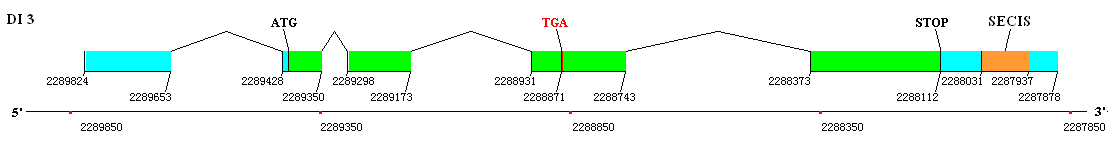


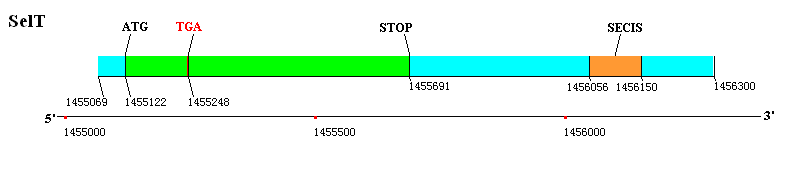


**Figure S2. Gene structures of SelN, Gpx a, Gpx b, Gpx d, Gpx e, Sel 15, DI 3 and SelT of *Ciona intestinalis*.**


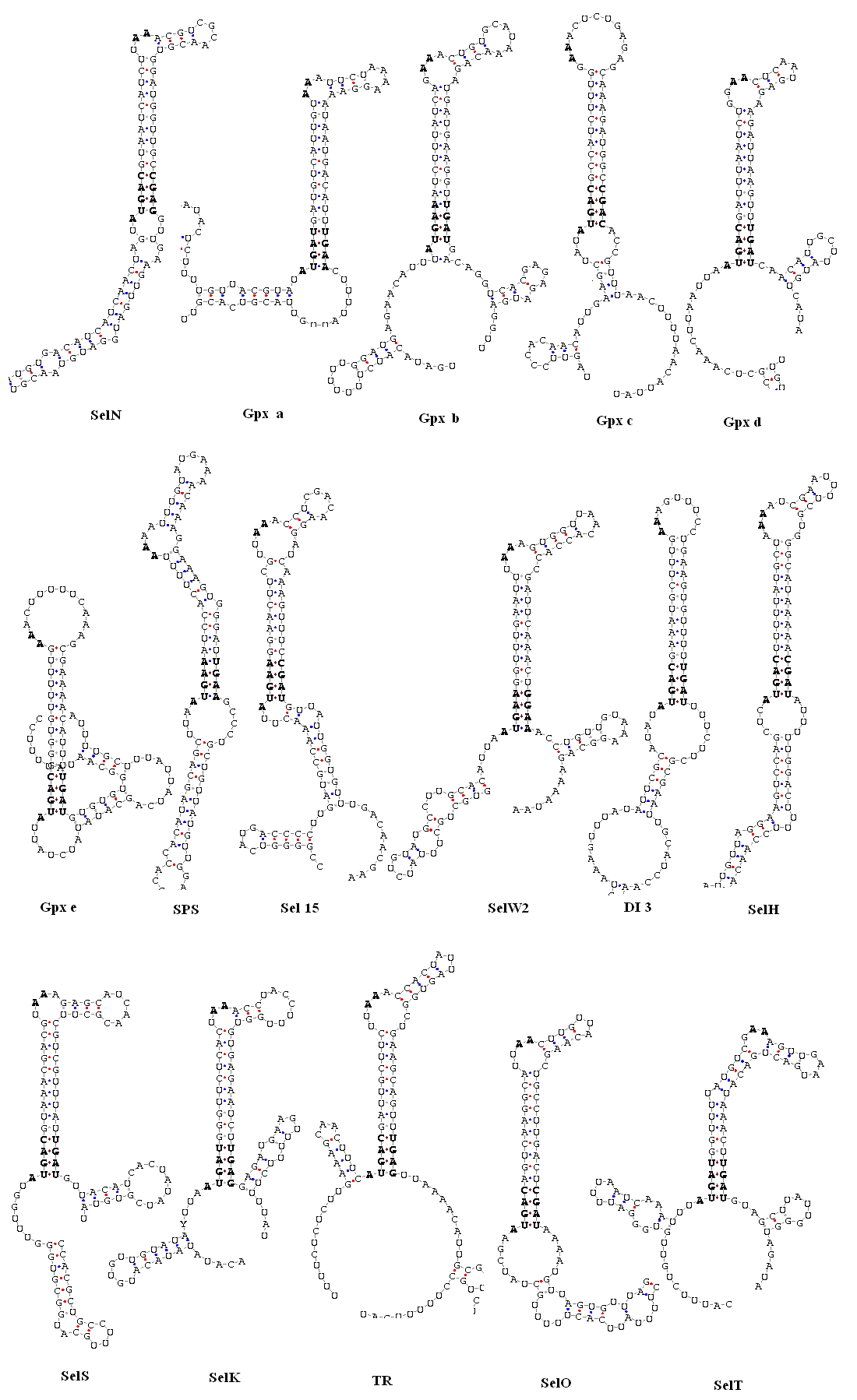


**Figure S3. Secondary structures of the SECIS elements of *Ciona intestinalis* selenoprotein genes.**


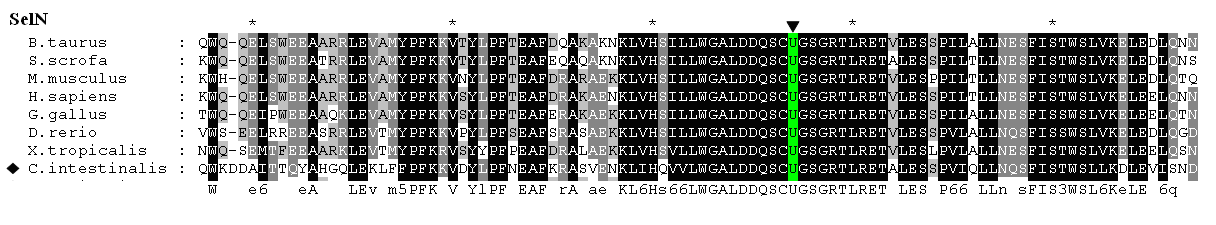


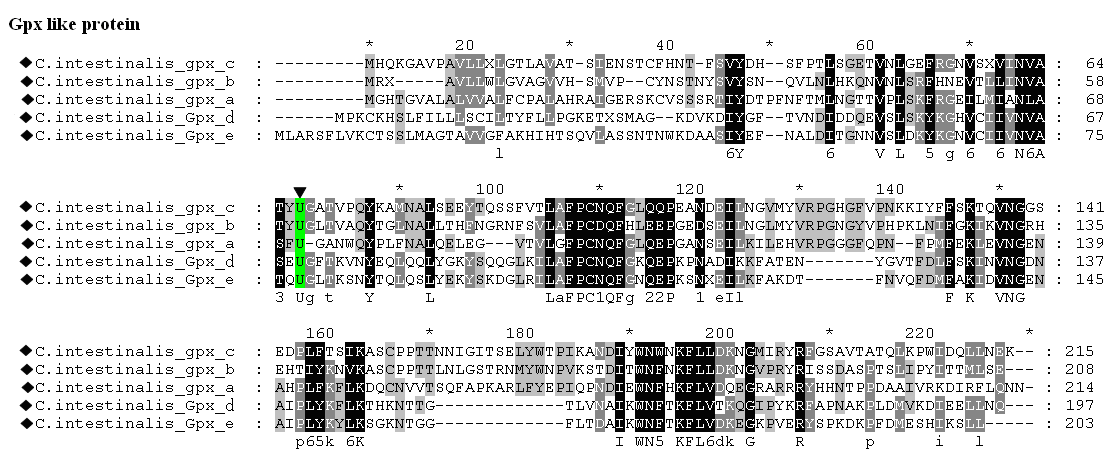


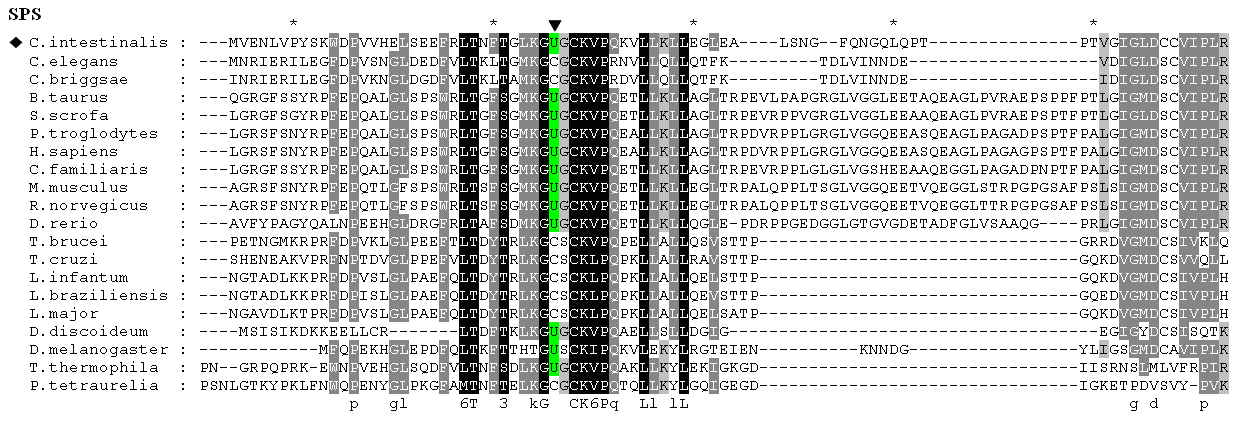


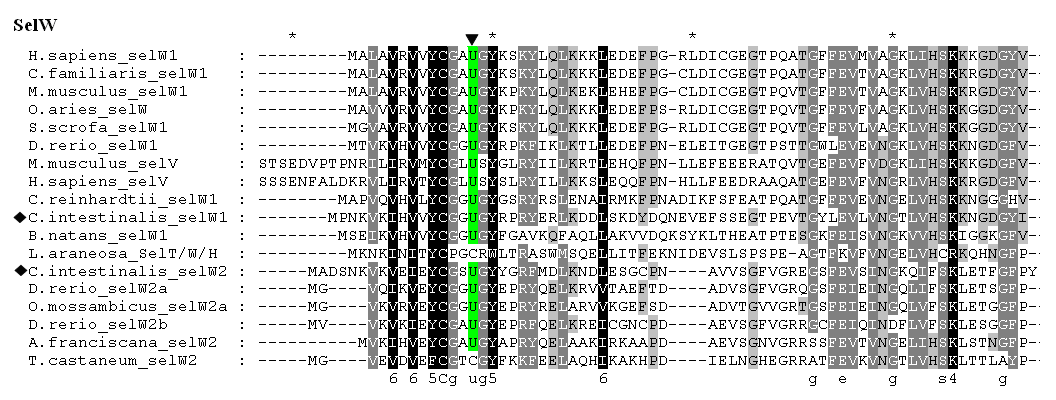


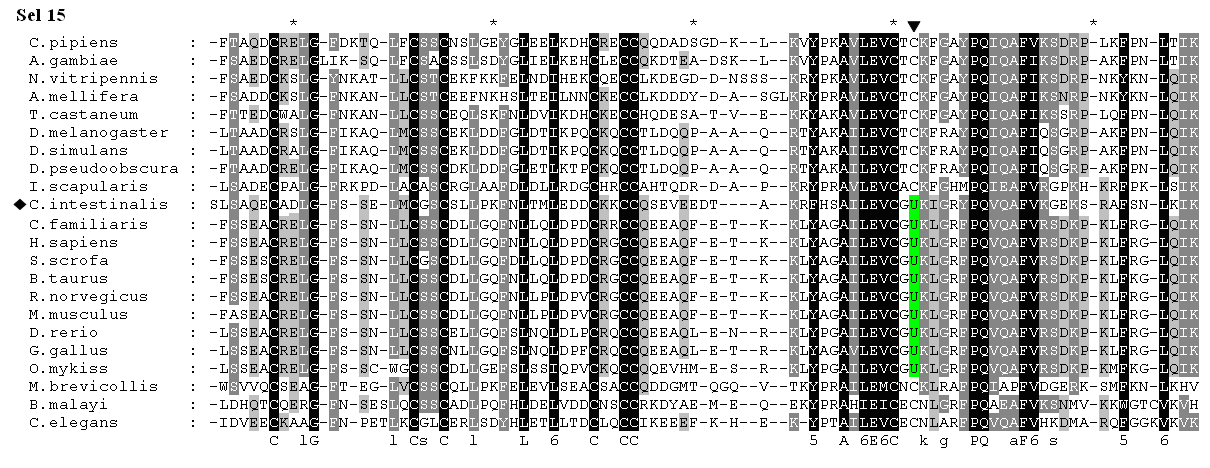


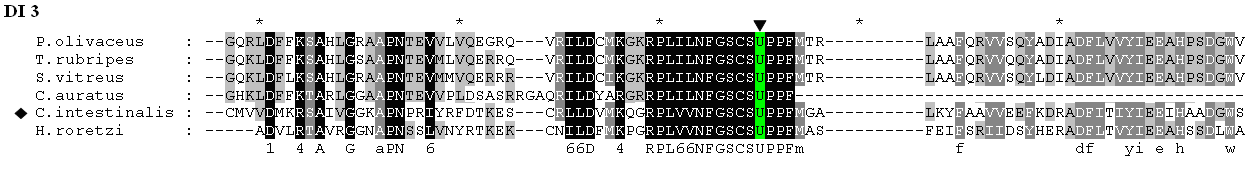


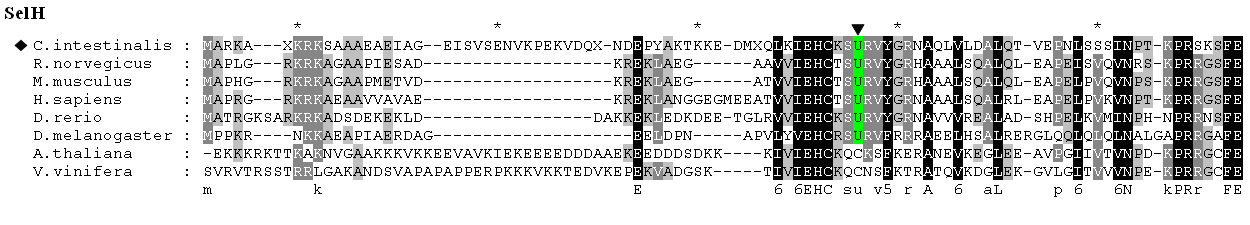


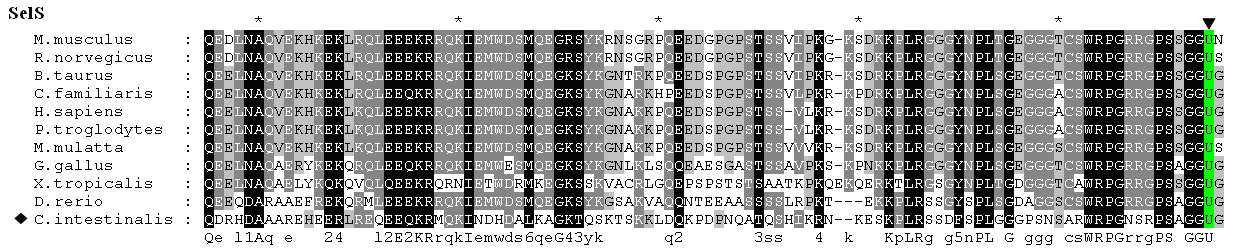


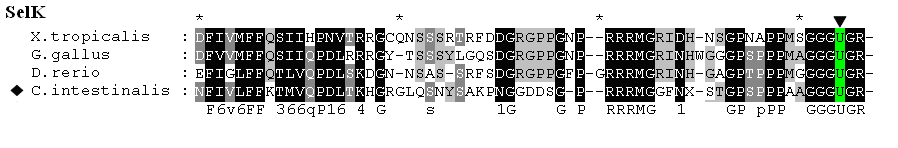


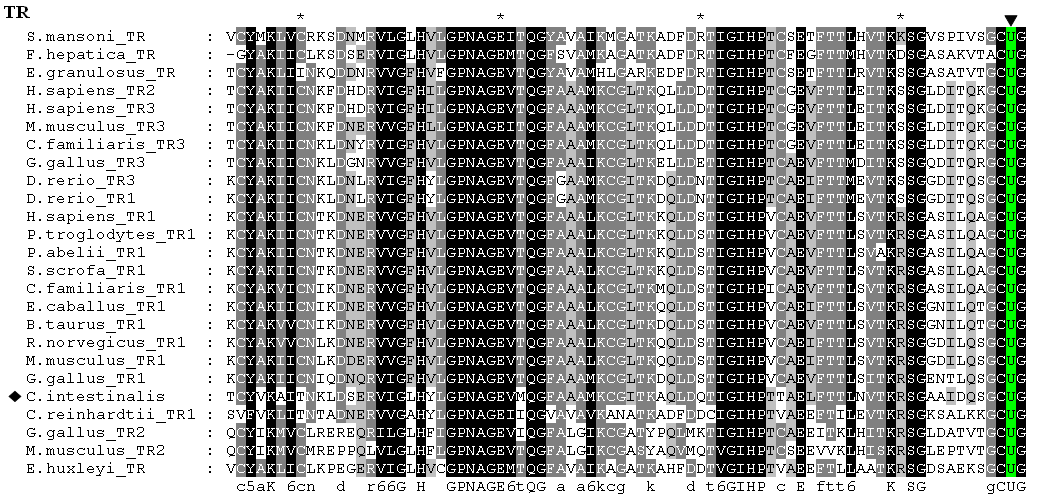


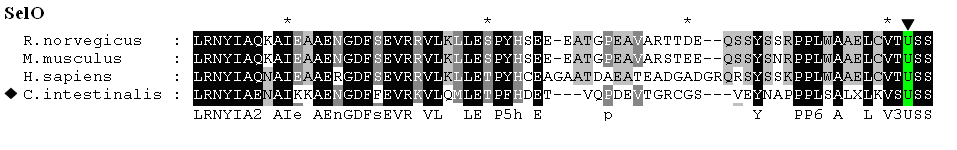


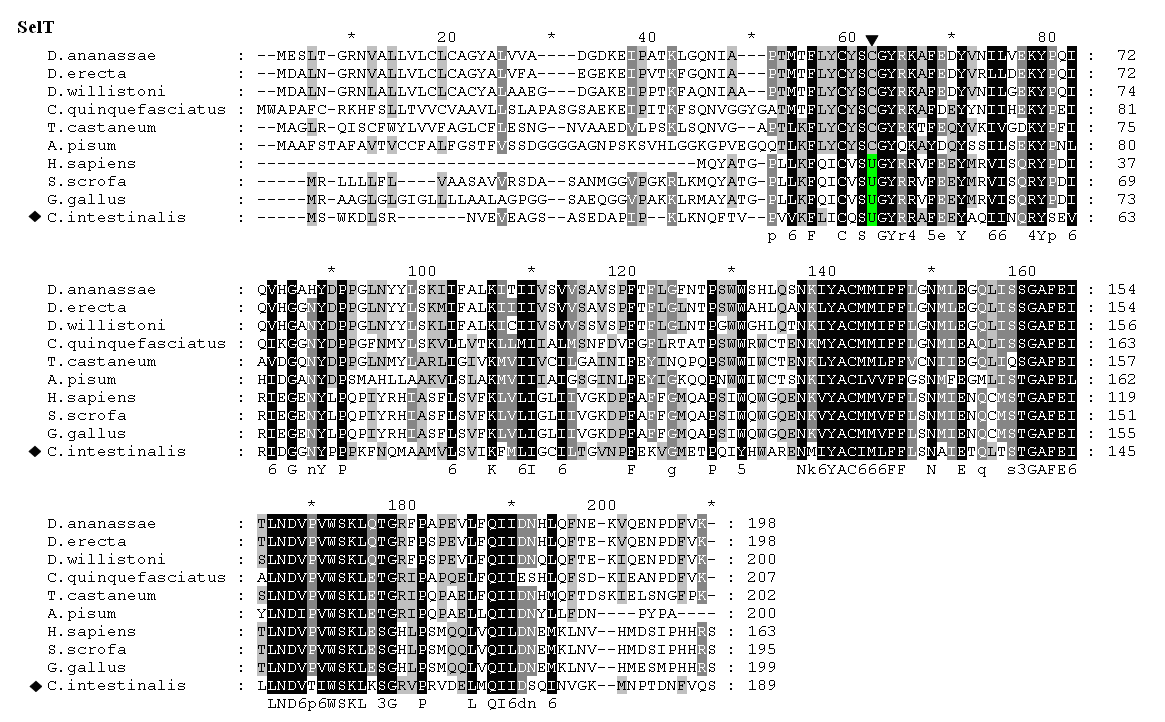


**Figure S4. Multiple alignments of newly identified selenoproteins and their homologous sequences.**

Species names of eukaryotes are listed on the left, newly identified selenoproteins are marked by the sign of ◆, and Sec residues are marked by ▼.


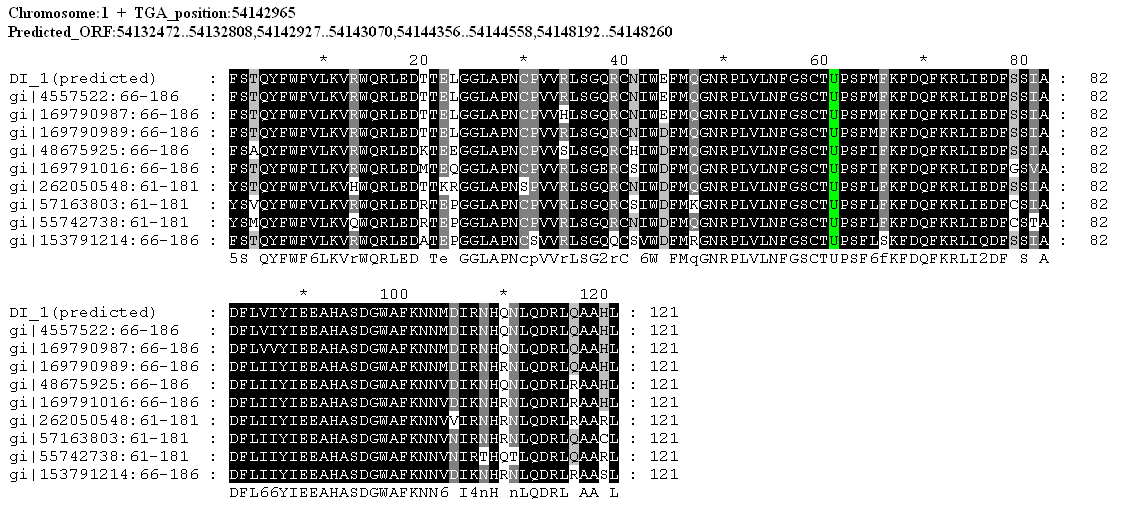


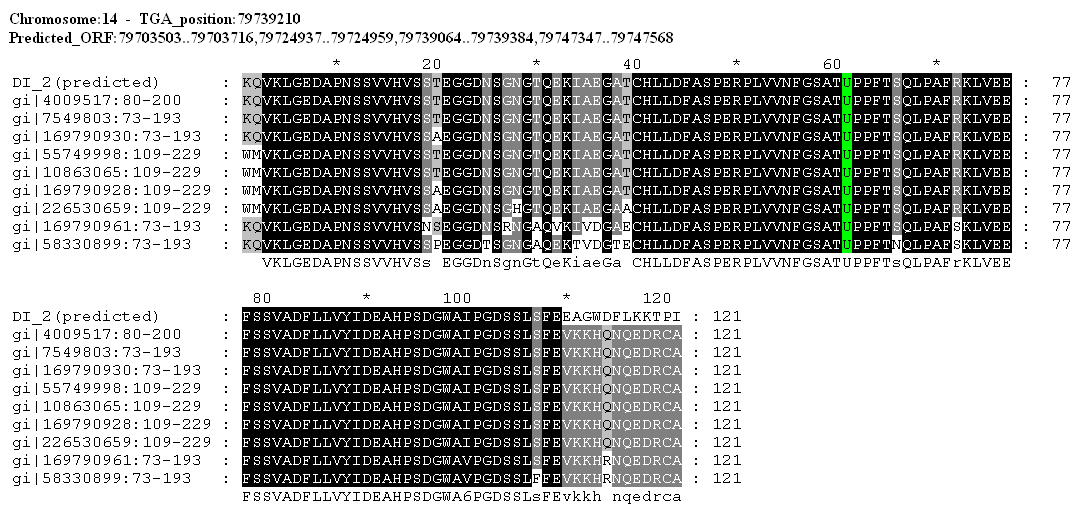

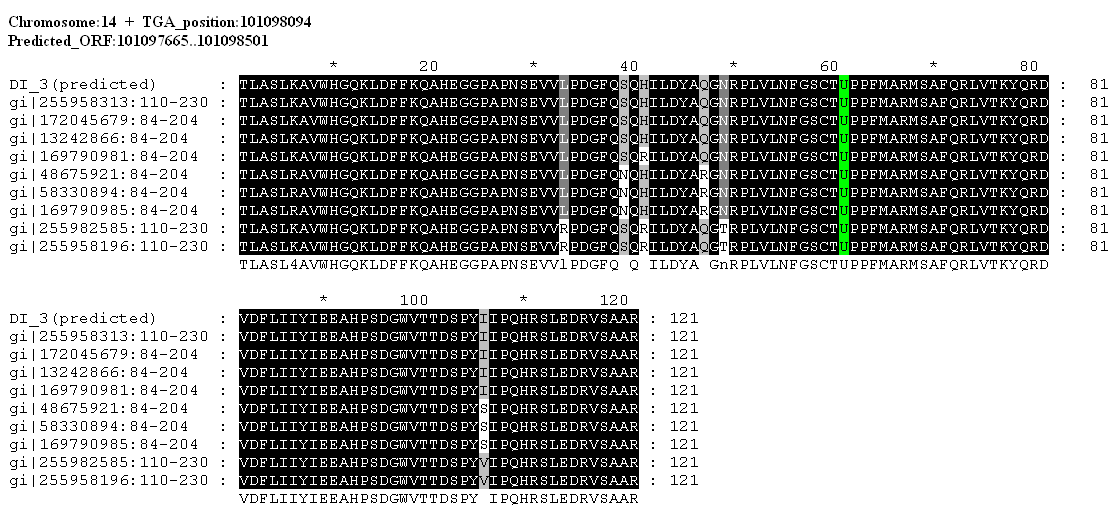

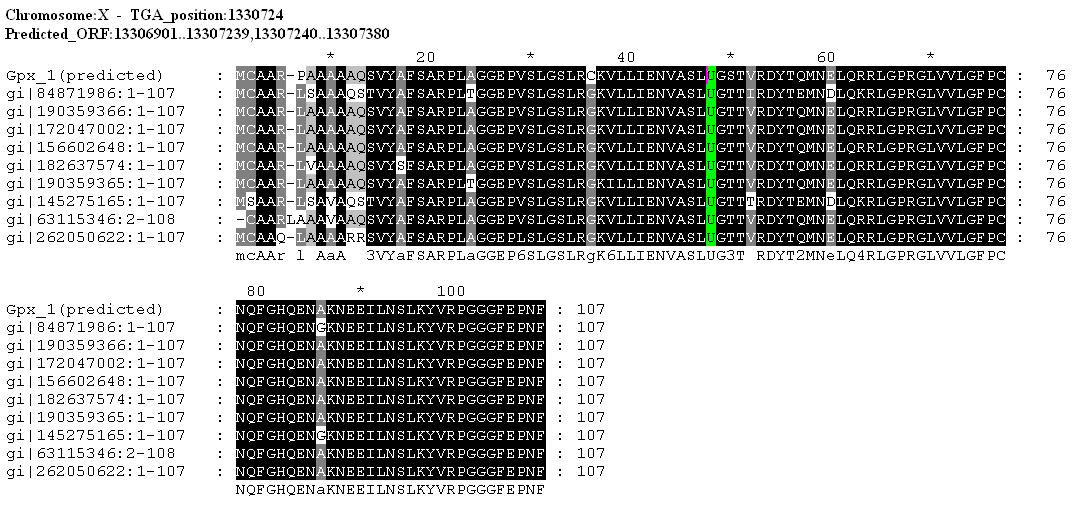

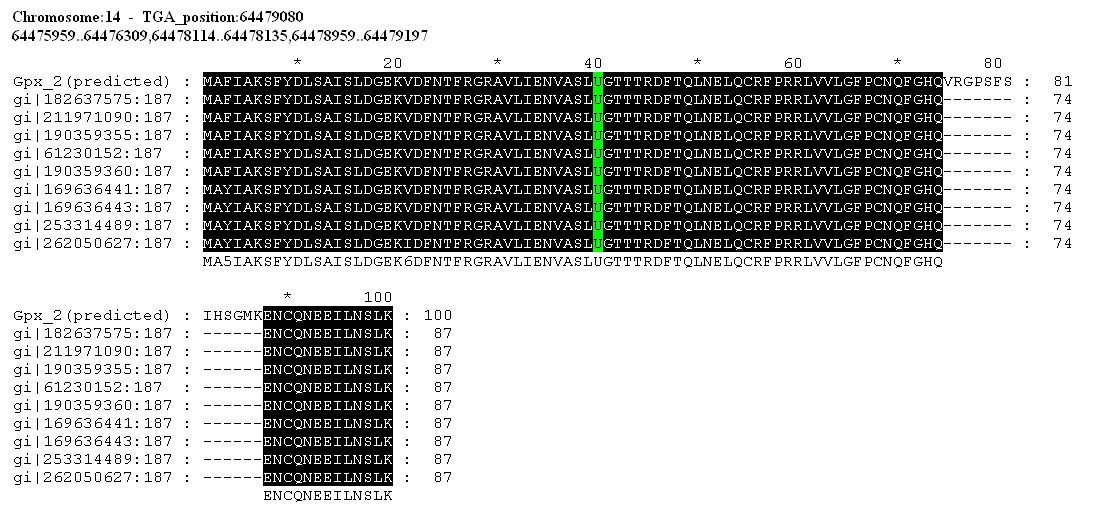

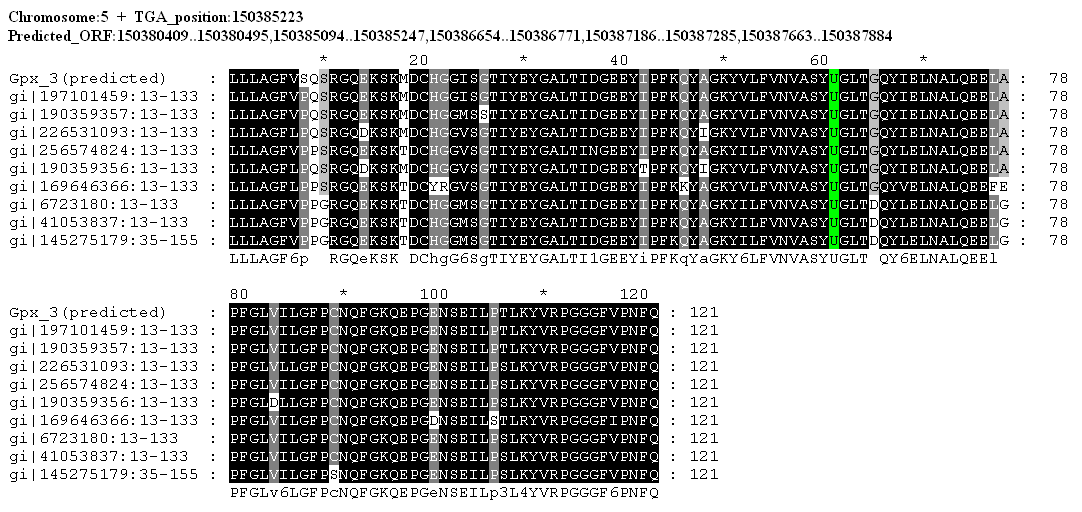

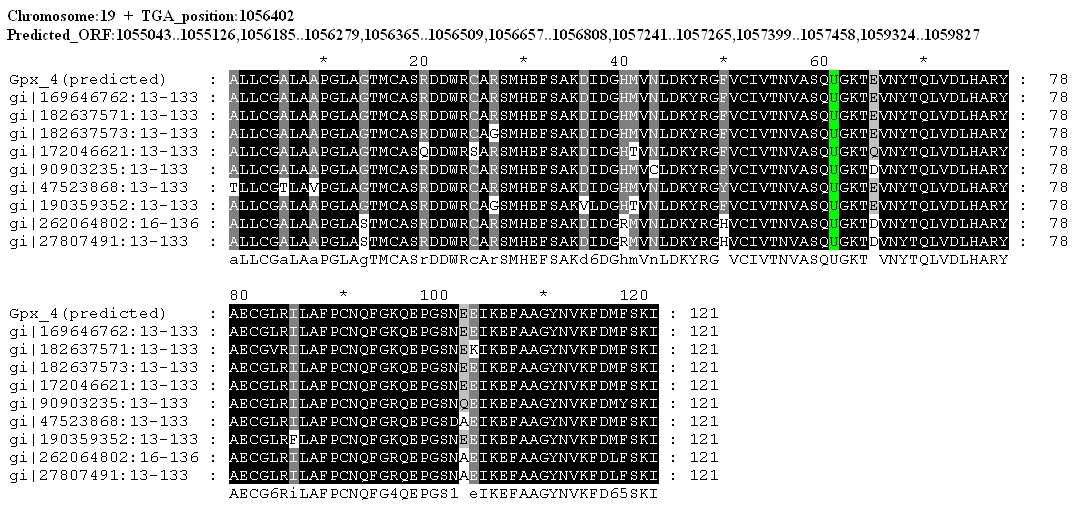

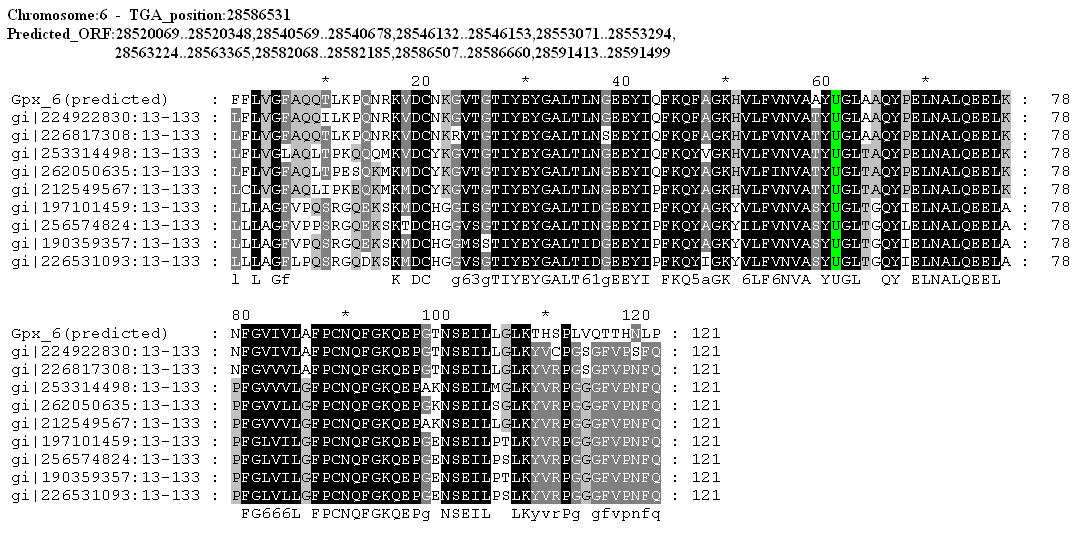

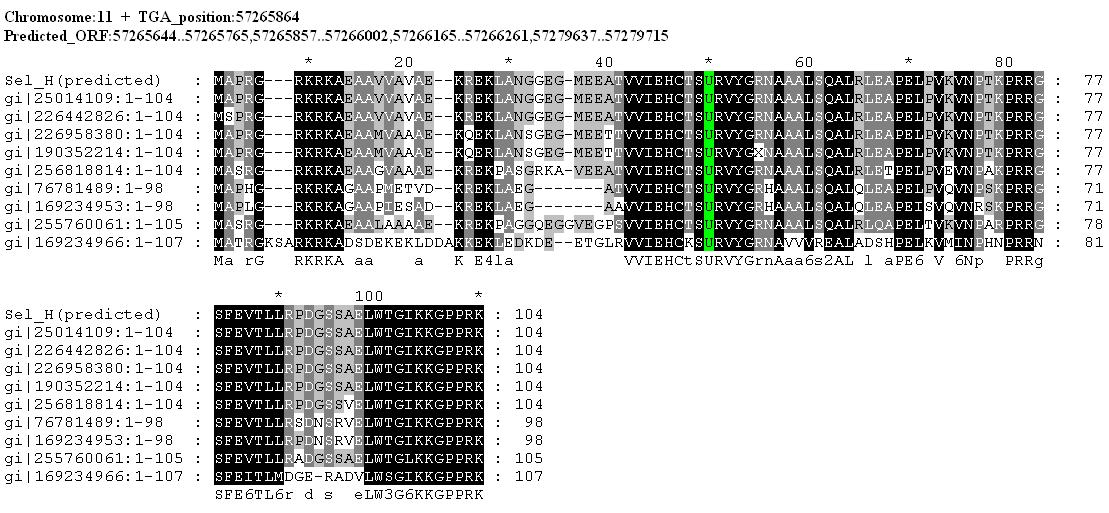

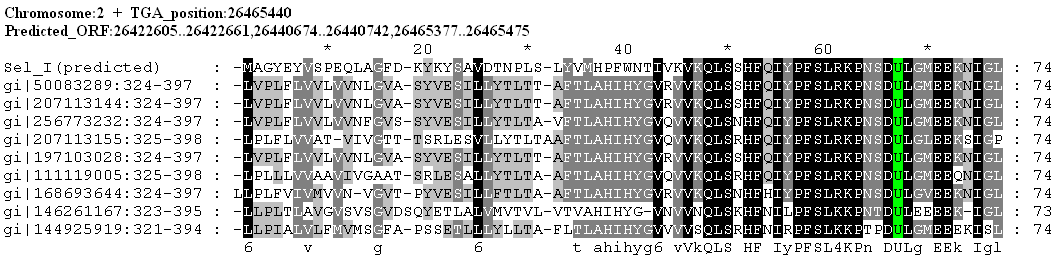

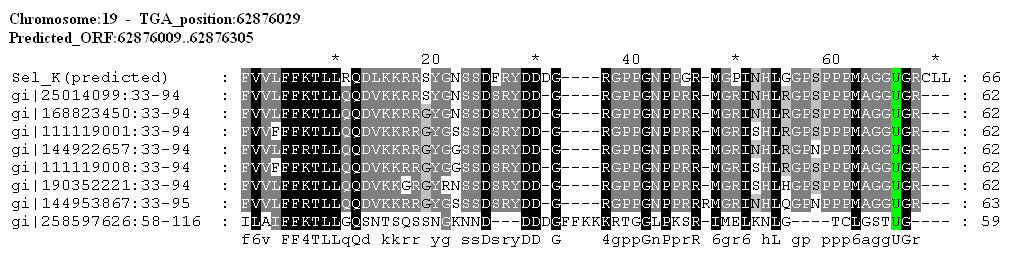


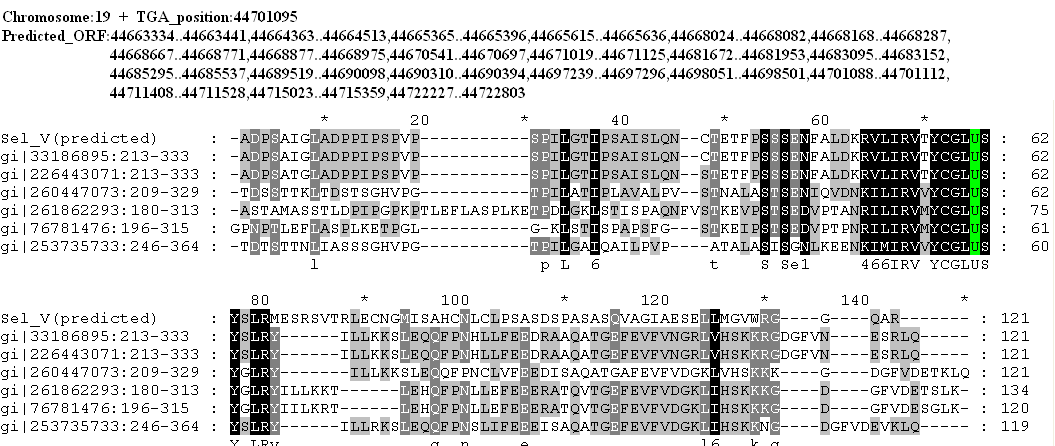

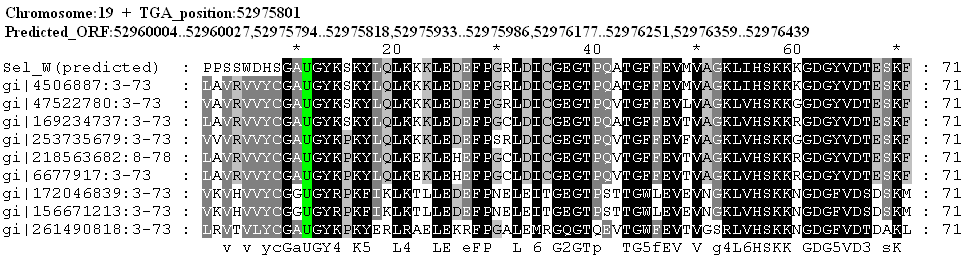

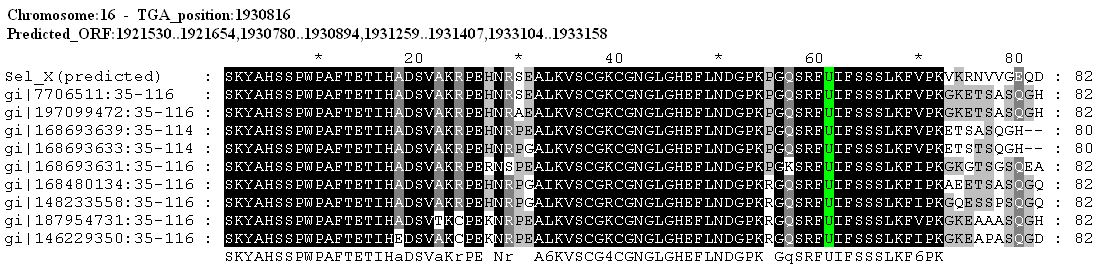

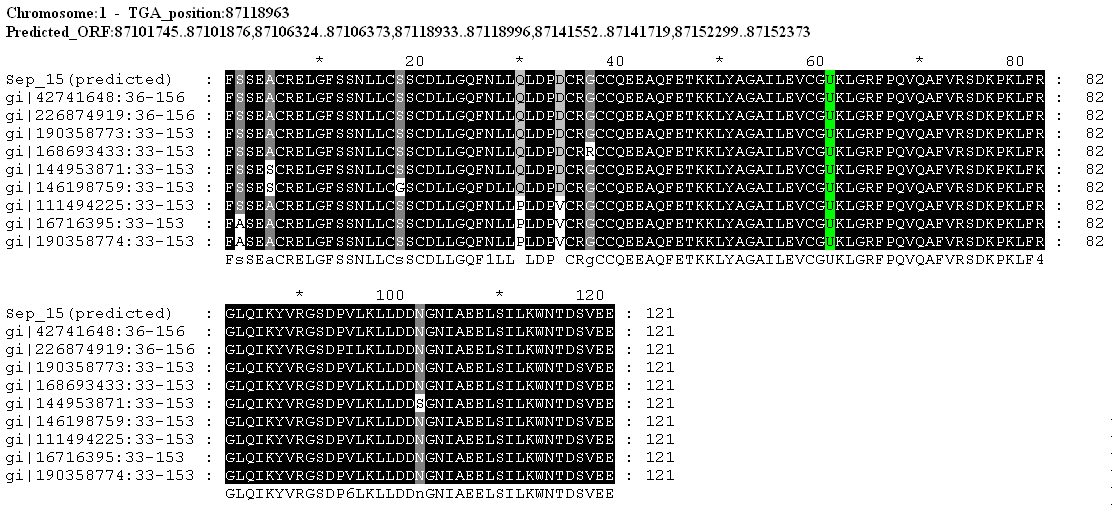

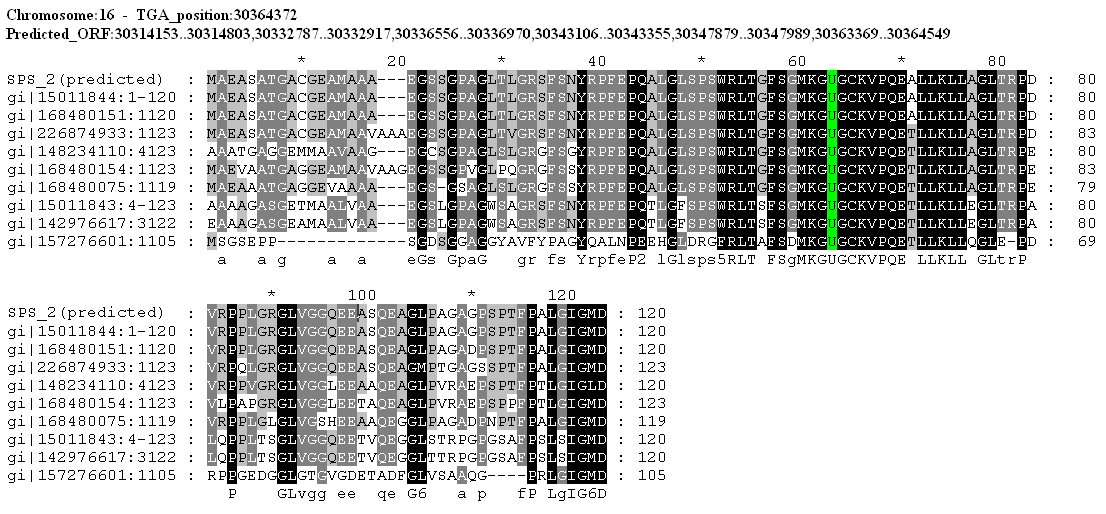

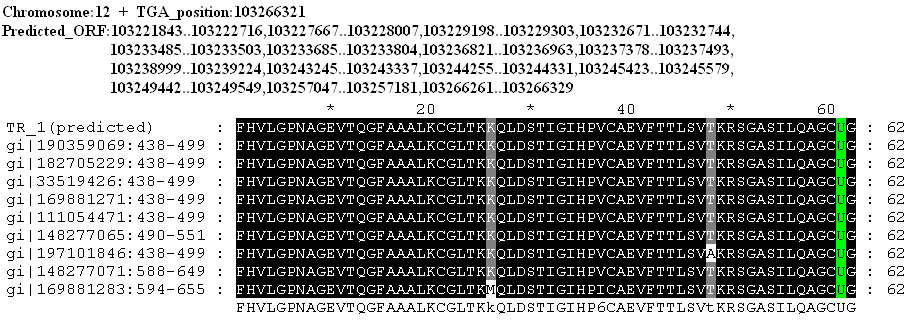

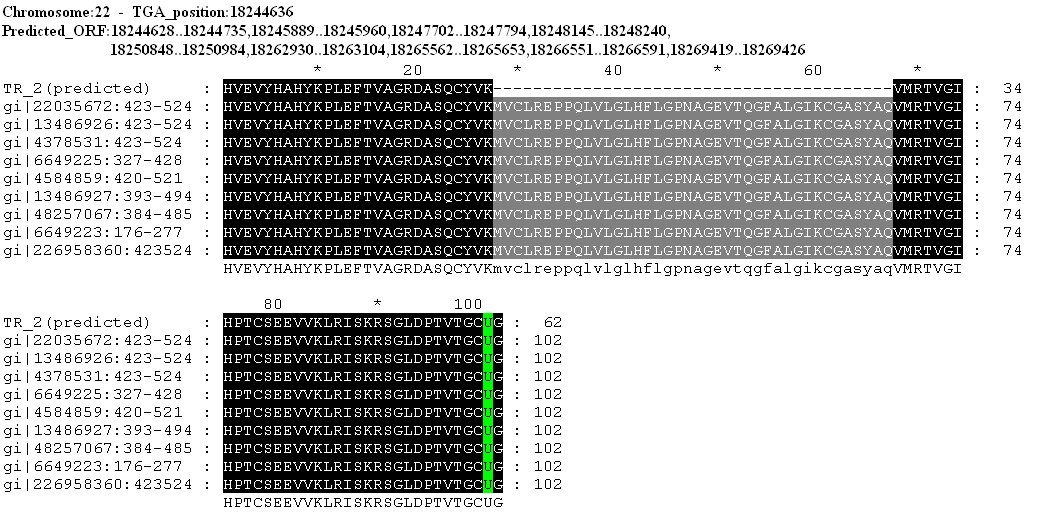

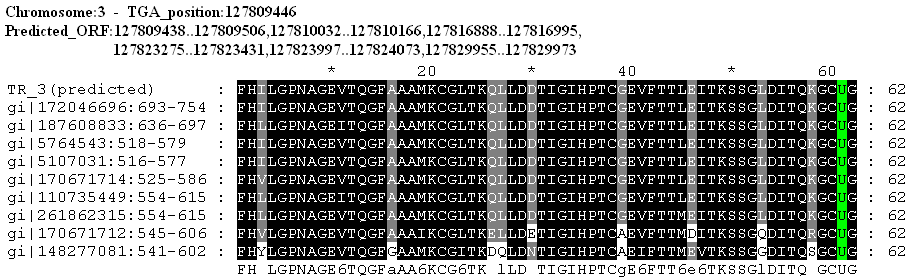

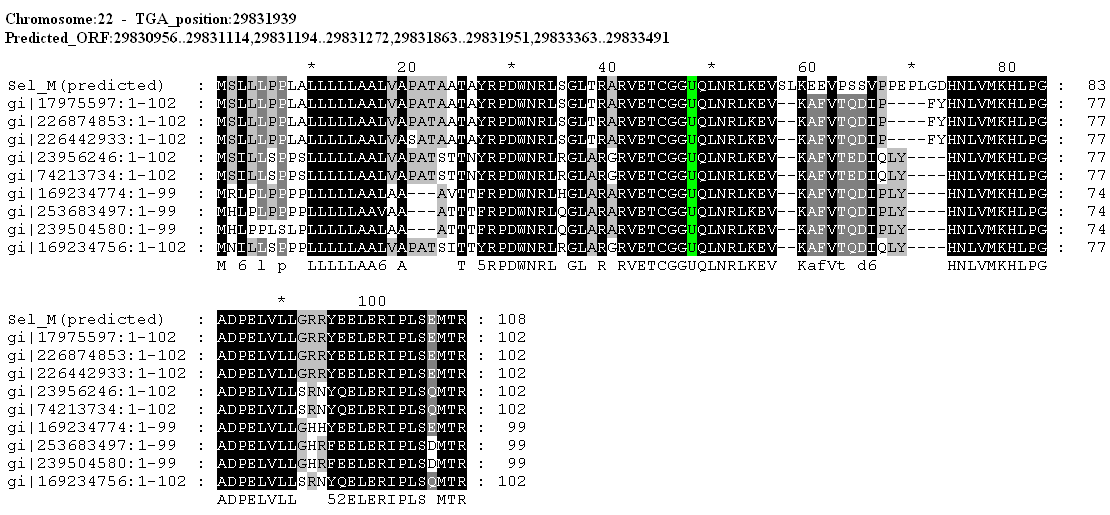

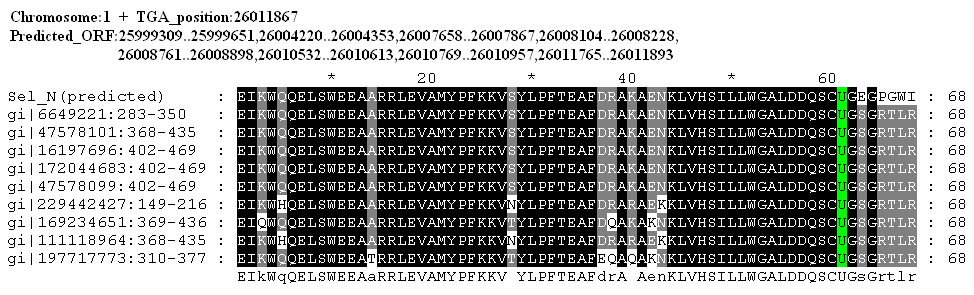

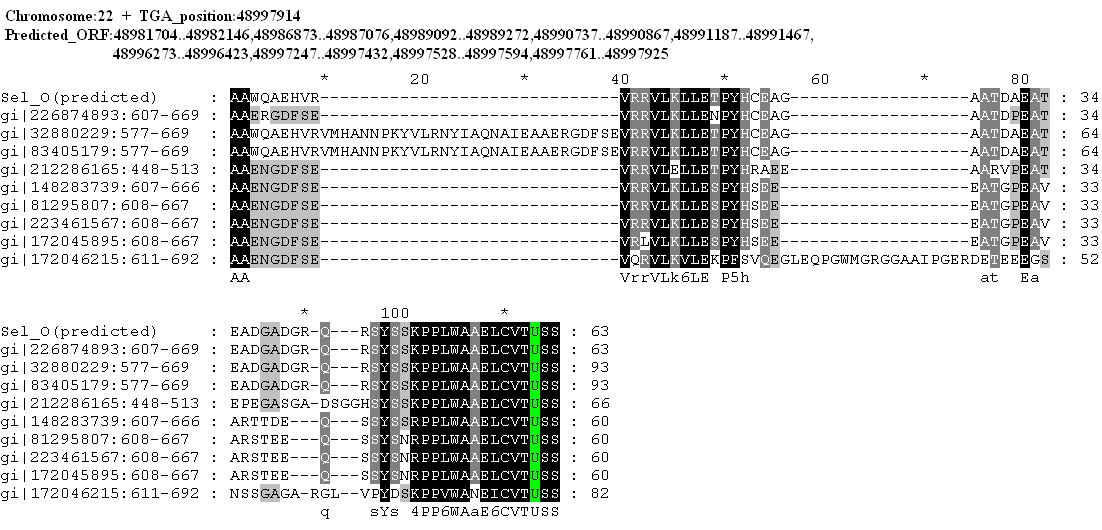

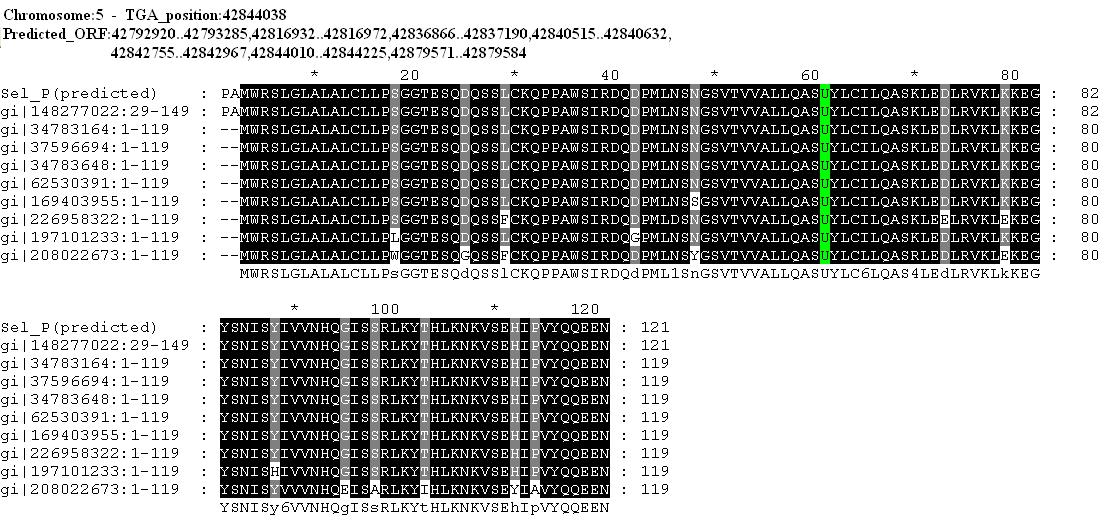

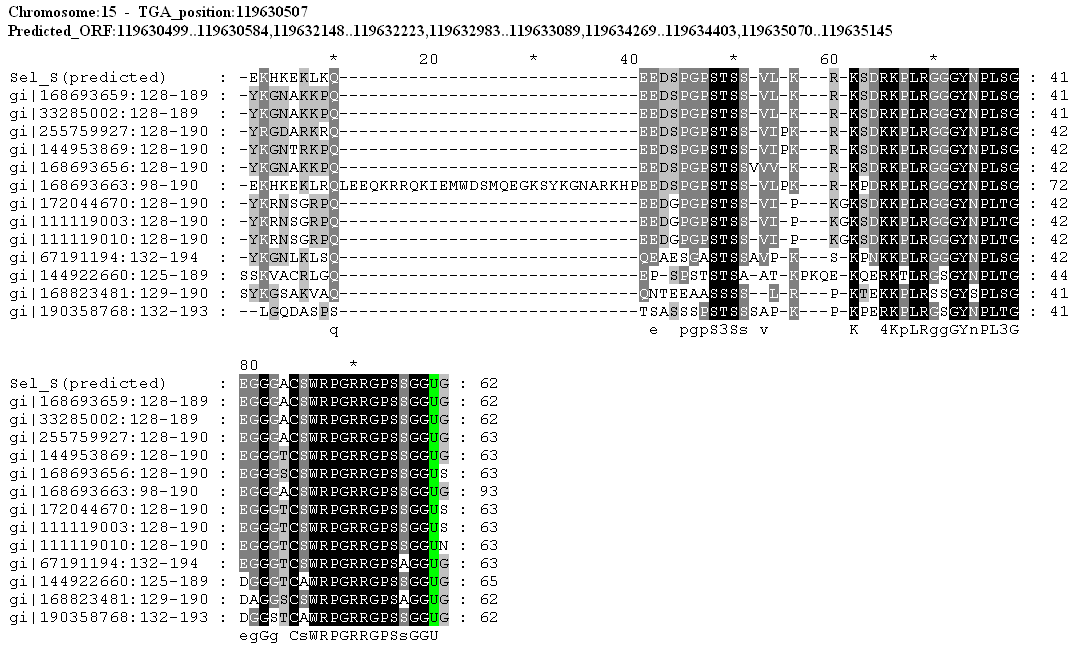

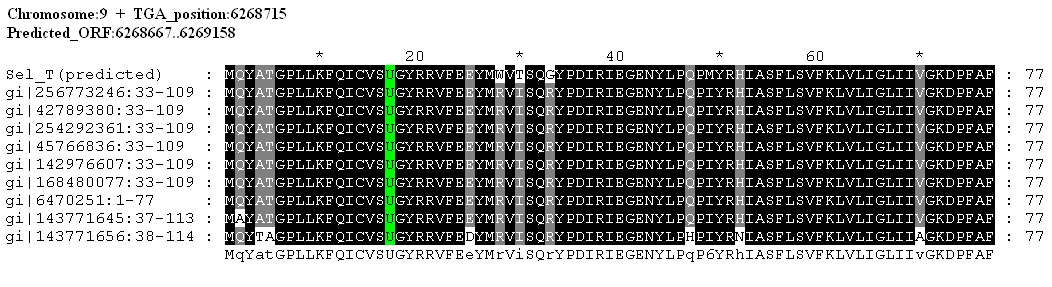


**Figure S5. Multiple alignments of human selenoproteins predicted via the SelGenAmic-based method and their homologous sequences.**

Information includes chromosome numbers, TGA and ORF positions of these genes. The gi numbers of the homologous sequences and the positions of the aligned parts are shown at the left side.


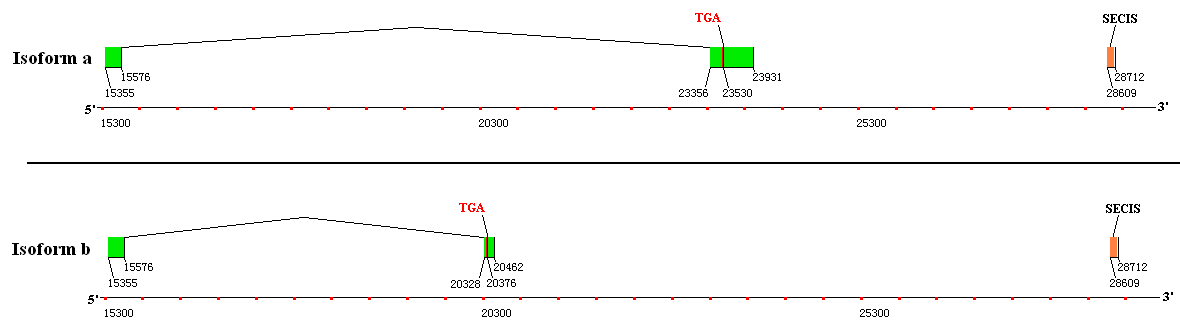


**Figure S6. Two alternative gene structures of the newly identified iodothyronine deiodinase type 2 (DI 2) from the *Dasypus novemcinctus* (armadillo) genome.**

The two selenoproteins were found in the scaffold_7983 of armadillo genome data available at the website: www.ensembl.org.


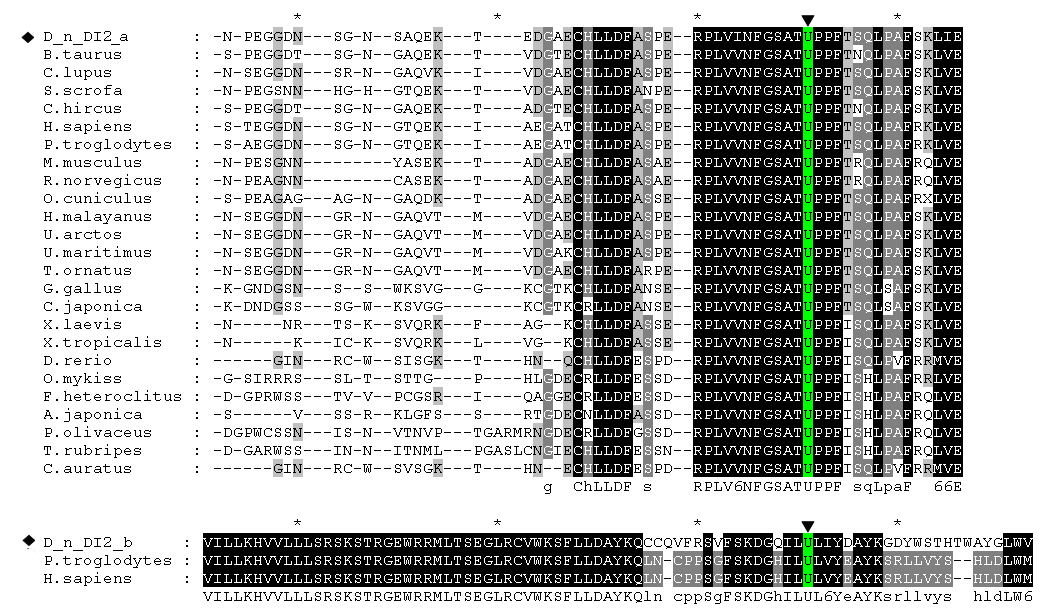


**Figure S7. Multiple alignments of the two newly identified *Dasypus novemcinctus* DI2_a and DI2_b genes and their homologous sequences.**

Species names of eukaryotes are listed on the left, newly identified selenoproteins of *Dasypus novemcinctus* are marked by◆, and Sec residues are marked by ▼.


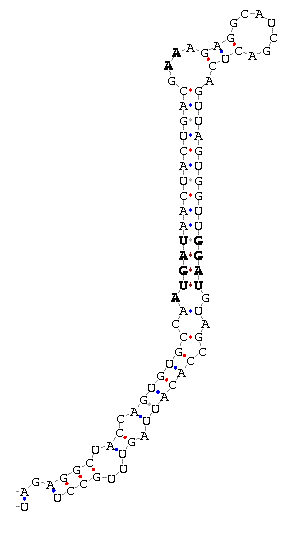


**Figure S8. Secondary structure of the SECIS element in *Dasypus novemcinctus* DI2_a and DI2_b genes.**
